# *cis*–*trans*-Amide isomerism of the 3,4-dehydroproline residue, the ‘unpuckered’ proline

**DOI:** 10.3762/bjoc.12.57

**Published:** 2016-03-29

**Authors:** Vladimir Kubyshkin, Nediljko Budisa

**Affiliations:** 1 Institute of Chemistry, Technical University of Berlin, Müller-Breslau-Str., 10, 10623, Berlin, Germany

**Keywords:** amino acids, *cis*–*trans* isomerism, fluorine, p*K*_a_, proline

## Abstract

Proline (Pro) is an outstanding amino acid in various biochemical and physicochemical perspectives, especially when considering the *cis*–*trans* isomerism of the peptidyl-Pro amide bond. Elucidation of the roles of Pro in chemical or biological systems and engineering of its features can be addressed with various Pro analogues. Here we report an experimental work investigating the basic physicochemical properties of two Pro analogues which possess a 3,4-double bond: 3,4-dehydroproline and 4-trifluoromethyl-3,4-dehydroproline. Both indicate a flat pyrroline ring in their crystal structures, in agreement with previous theoretical calculations. In solution, the peptide mimics exhibit an almost unchanged equilibrium of the *trans*/*cis* ratios compared to that of Pro and 4-trifluoromethylproline derivatives. Finally we demonstrate that the 3,4-double bond in the investigated structures leads to an increase of the amide rotational barriers, presumably due to an interplay with the transition state.

## Introduction

**Scheme 1:**
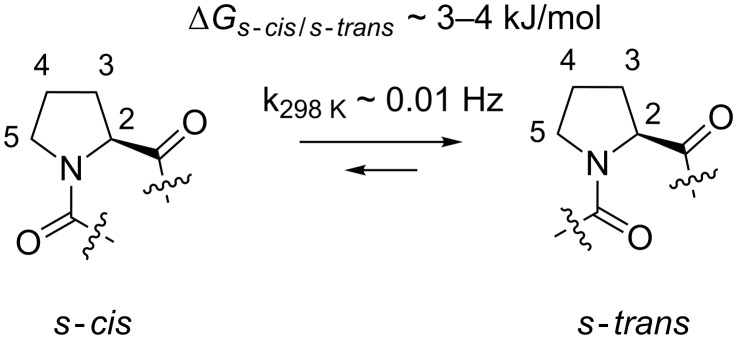
s-*cis*–s-*trans* Amide bond isomerism in an *N*-acyl-proline fragment.

The sole genetically encoded secondary amino acid proline (Pro, **1**) is known for its unique properties in biological systems. In particular, Pro residues are often found in the *s*-*cis* peptidyl-Pro conformation, due to the low energy difference between the *s*-*trans* and the *s*-*cis* conformational states (ca. 3–4 kJ/mol) [1–2]. In addition, the high energy barrier of the s-*cis*–s-*trans* isomerization (84–89 kJ/mol) stabilizes the amide conformers kinetically (Scheme 1) [3]. By comparison of the amide rotational rates of peptidyl-Pro with the ones of the closest Pro structural analogues, azetidine-2-carboxylic acid (norproline) and pipecolic acid (homoproline) [4], it appears that the high isomerization barrier is a feature associated with the 5-membered pyrrolidine ring of Pro [5].

The pyrrolidine ring of Pro can be found in several conformations, designated as the *exo*- and *endo*-puckers, as well as in twisted forms [6–7]. Various ring substituents can significantly shift the equilibrium towards a high preference for one particular conformation. For instance, 3- [8–9] and 4-ring [10–15] substituents have been characterized to shift the pucker equilibrium, and as such adversely affect the s-*trans*/s-*cis* equilibrium preferences of the amide bond. Conversely, substitutions in the ring positions 2 [16–18] and 5 [19–21] shift the amide equilibrium towards higher contents of s-*trans* and s-*cis* forms, respectively, due to the steric reasons. However, it has also been reported that *N*-acylated pyroglutamic acid exhibits almost exclusively the s-*trans* amide conformation despite being formally a 5-substituted Pro [22]. The conformational preferences of the heterocyclic analogues of proline [23–25] and, in particular, pseudoprolines [26–28] have also been characterized.

**Figure 1:**
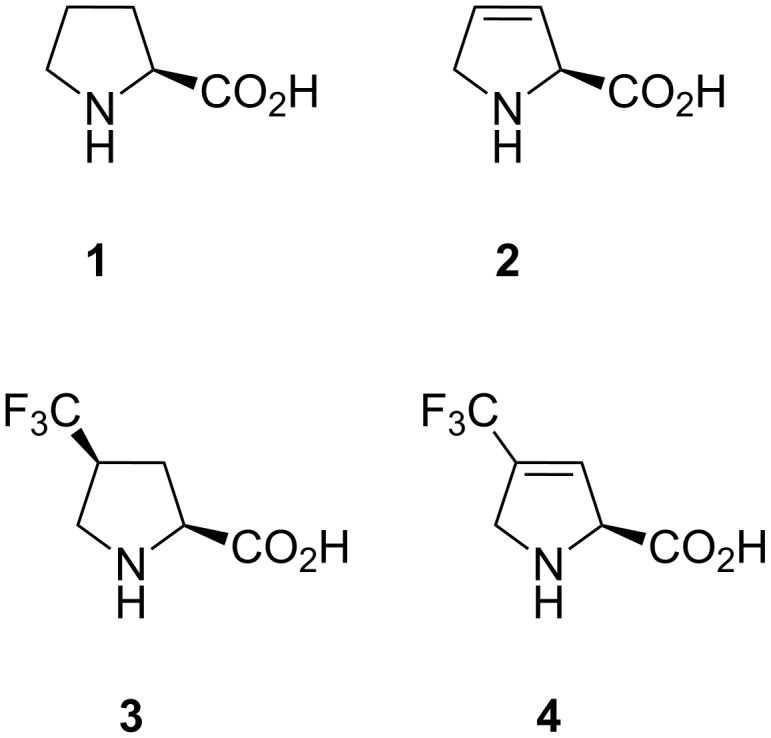
Amino acids that have been compared in this study.

3,4-Dehydroproline (Dhp, **2**, Figure 1) has been reported to exhibit a rather flat ring structure, as the result of theoretical analysis of Ac-Dhp-NHMe models [29–30]. It has also been demonstrated that Dhp inhibits collagen biosynthesis and suppresses the hydroxylation of proline [31–33]. Recently we found, in a comparative study of proline analogues, that Dhp is a translationally active amino acid, which, when compared to proline, exhibited lower rates of translation [34]. In order to further understand the role and potential of Dhp, this amino acid requires parametrization of its basic physicochemical features. Previously, in the literature the experimental characterization of Dhp has not been properly discussed. Herein we report the NMR and crystallographic characterization of Dhp (**2**) and 4-trifluoromethyl-3,4-dehydroproline (TfmDhp, **4**) in simple models. Data on Pro (**1**) and (4*S*)-trifluoromethylproline (**3**) is used for comparison.

## Results and Discussion

Firstly, we determined the p*K*_a_ of the ammonium group in the free amino acids (Table 1). Overall the values demonstrate a 0.9 p*K*_a_ reduction upon introduction of the 3,4-double bond, and a 2.2 p*K*_a_ reduction for the 4-CF_3_-group. Thus, both modifications lead to electron depletion of the ring system. In addition, the larger effect upon incorporation of the CF_3_-group indicates that both analogues **3** and **4** possess a similar orientation of the side-chain substituent with respect to the amine group. Previously we speculated that the CF_3_-group in **3** should adopt an equatorial conformation, and thus stabilize the *exo*-pucker, based upon considerations of the vicinal *J*-couplings (in Ac-TfmPro-OMe, **7**) [35]. Considering that the CF_3_-substituent should be located within the plane of the 3-pyrroline ring in **4**, this, indeed, has an orientation close to the equatorial CF_3_-group placement in **3**, and is not axial.

**Table 1:** p*K*_a_ Values determined for the amino acids **1**–**4** and their *N*-acetyl derivatives.

Xaa	ammonium group p*K*_a_^a^	carboxyl group p*K*_a_^a^		
	in Xaa	in Ac-Xaa		
		*s*-*trans*	*s*-*cis*	Δp*K*_a_
	
**1**, Pro	10.68	3.55	2.85	0.70
**2**, Dhp	9.78	3.03	2.37	0.66
**3**, TfmPro	8.46	3.21	2.57	0.64
**4**, TfmDhp	7.60	2.65	1.99	0.66

^a^In aqueous medium at 298 K, standard error for the amino group ±0.10, for the carboxyl group ± 0.05. Δp*K*_a_ = p*K*_a_ (*s*-*trans*) – p*K*_a_ (*s*-*cis*).

Next, we determined the p*K*_a_ of the carboxyl groups in *N*-acetyl amino acids for two rotameric forms separately (Table 1). All four compounds exhibited similar Δp*K*_a_ values [36–37]. Though, absolute acidity was depressed with both the 3,4-double bond and 4-CF_3_ substitutions, the former had a stronger impact. Thus it is evident that the 3,4-double bond significantly increases the electrophilicity of the carbonyl group of the amino acid residue.

The effect on the *s*-*trans*/*s*-*cis* equilibrium was revealed upon NMR investigations of the conventional methyl esters of the *N*-acetyl amino acids (Ac-Xaa-OMe) [38]. The equilibrium *K*_s-_*_trans_*_/s-_*_cis_* constants in the model compounds were found to be: **5** – 4.97 ± 0.07, **6** – 5.45 ± 0.09, **7** – 4.31 ± 0.05 and **8** – 4.82 ± 0.03 (50 mM, D_2_O, 296 K). In terms of the free energy the 3,4-double bond increased the relative stability of the s-*trans* conformer by 0.2–0.3 kJ/mol, whereas the 4-CF_3_-group demonstrated an opposite effect of about 0.3 kJ/mol (standard error ±0.1 kJ/mol). Despite both effects being rather marginal, this indicates that the increase of the electrophilicity of the terminal carbonyl groups (as seen previously in Ac-Xaa acidity) does not have a significant impact on the intramolecular interaction between the two carbonyl groups (as seen from *K*_s-_*_trans_*_/s-_*_cis_* values). Similarly, Jenkins et al. reported on bicyclic proline analogues and demonstrated that axially oriented electron withdrawing substituents (4-fluoro and 4-hydroxy groups) maintained the *K*_s-_*_trans_*_/s-_*_cis_* equilibrium values of the parent unsubstituted structure [39].

**Figure 2:**
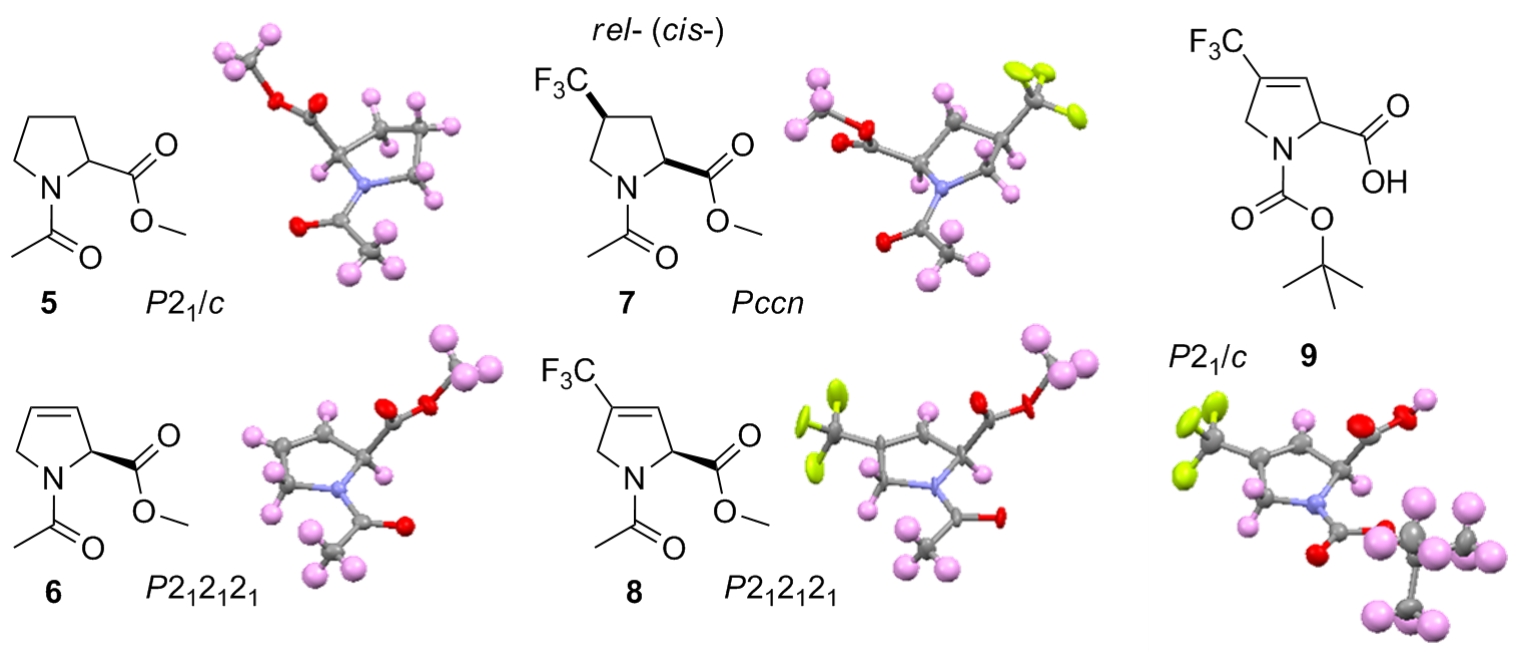
X-ray crystal structures of compounds **5**–**9**. Carbon- grey, nitrogen – blue, oxygen – red, fluorine – yellow, hydrogen – purple.

We also analyzed X-ray crystal structures of the Ac-Xaa-OMe models. All four compounds crystalized in the s-*trans* conformation: **5** and **7** as racemates [40], **6** and **8** as single enantiomers (Figure 2). In addition, *rac*-Boc-TfmDhp (**9**) crystals were subjected for analysis as well. The latter crystal structure demonstrated a pseudo *s*-*cis* conformation due to the helical hydrogen bond structure established between the free carboxylic and the carbamate groups as C(=O)–O–H···O=C(–O*t*-Bu)–N. The same has been recently reported in particular in the cases of *N*-Boc-2-methylproline [41] and *N*-Boc 4,5-difluoromethanoproline [42].

In the crystal structure the ring conformations were found to be: twisted *endo*-pucker for **5**, *exo*-pucker for **7**, and compounds **6**, **8** and **9** exhibited reasonably flat pyrroline rings, that is in agreement with previous computational works. (Alternative ‘flattened’ proline analogues – 4,5-methanoprolines have also been characterized, see [43–44].) For **6** and **8** the φ-angles were found to be −69 and −67° respectively, which are typical values for a Pro residue. Importantly, both structures did not indicate any pyramidalization around the amide nitrogen atom. The N–C=O→C=O(OMe) angle was 94–97°, which is below the optimal Bürgi–Dunitz trajectory angle [45–46]. The φ-angles in **6** and **8** found in the crystals and the *K_s-trans_*_/s-_*_cis_* values found in solution both indicate close conformational similarities between Dhp and Pro fragments.

**Scheme 2:**
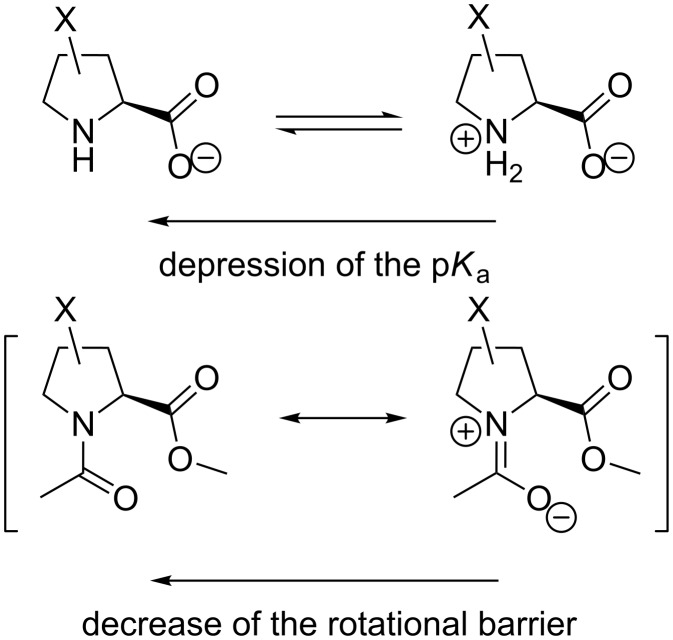
The relationship between the p*K*_a_ of the ammonium function in the amino acid and the amide rotational barrier in proline analogues. The substituents that impose a p*K*_a_ depression effect should also decrease the content of the resonance structure with the separate charges in the ground state of the corresponding amide, that leads to a lowering of the rotational barrier.

**Figure 3:**
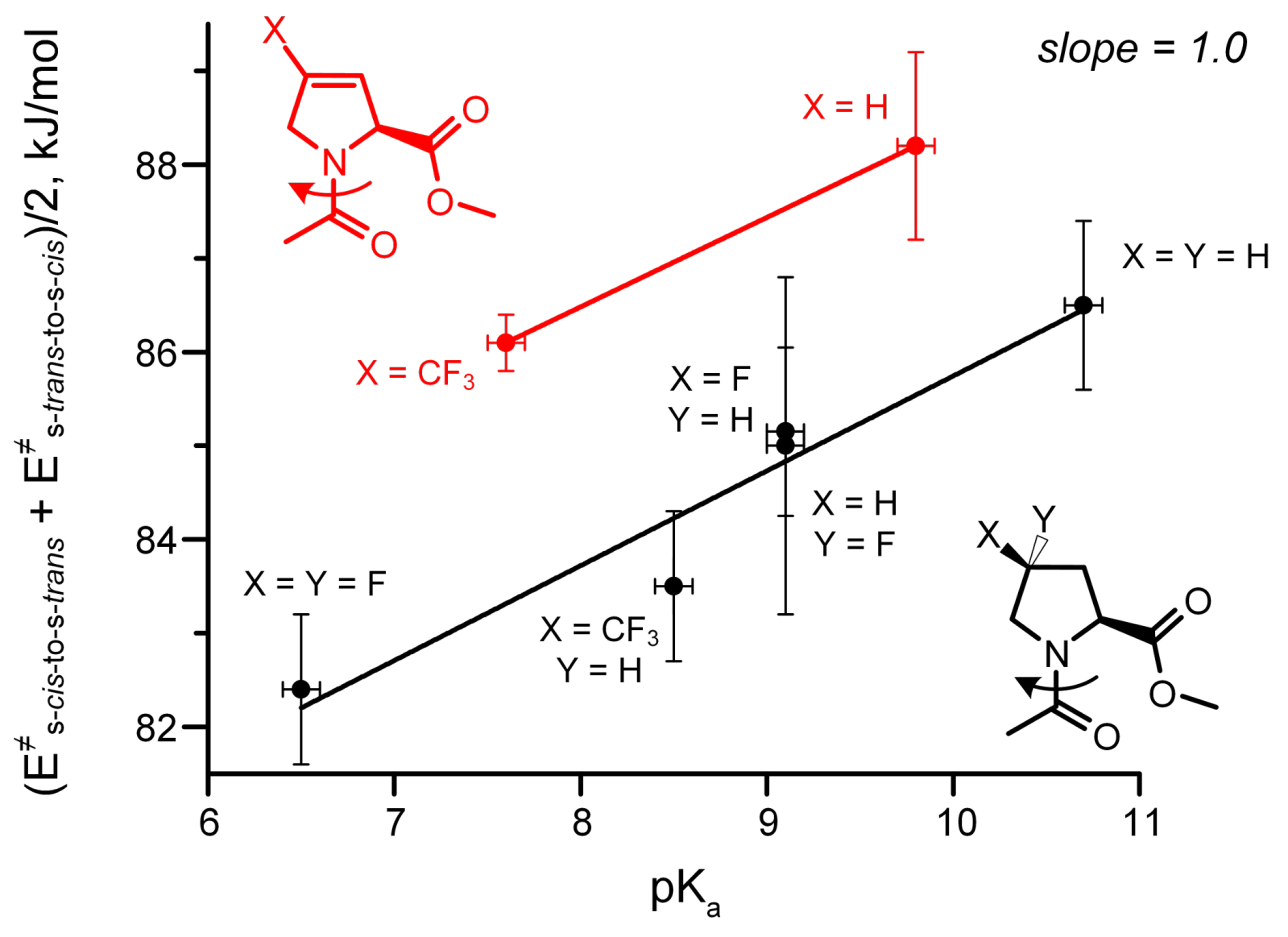
The double bond between C^3^ and C^4^ atoms in 3,4-dehydroproline residues induces an increase in the amide rotational barriers in Ac-Xaa-OMe.

Finally, we identified the amide rotation rates and corresponding activation barriers by EXSY NMR (D_2_O, 310 K). In order to establish a proper reference system we correlated the observed activation energies with the p*K*_a_ of the ammonium groups in the corresponding free amino acids (Scheme 2). The resulting correlation indicates a remarkable offset in the activation energy in the 3,4-double bond containing residues (Figure 3). This effect was not only observed in water, but also persisted in organic solvents (MeOD, DMSO, CDCl_3_, see Supporting Information File 1). The activation energies are thus generally increased by the presence of the double bond by ca. 2.7 kJ/mol, that corresponds to about a factor of 1/3 of the rotational rates.

Considering that no nitrogen pyramidalization has been observed in the crystal structures, this would indicate a destabilization of the transition state by the 3,4-double bond in Dhp and TfmDhp. Indeed, it is well known, that the peptidyl-Pro amide bond rotation proceeds via the *syn*/*exo* transition state, where the oxygen atom of the amide group moves under the pyrrolidine ring, approaching to C^3^ and C^4^ atoms [47]. Repulsion between the oxygen lone pairs and the double bond in the Dhp residue could cause the experimentally evident increase in the rotation barriers.

## Conclusion

In summary, we performed the experimental characterization of proline analogues with a 3,4-double bond: Dhp and TfmDhp. Our results confirmed ‘flattening’ of the proline ring by the double bond, in agreement with what has been previously suggested by theoretical studies. Both, the 3,4-double bond and the 4-CF_3_-group impose electron-withdrawing effects on the functional groups of the amino acids. Though, the carboxyl function is influenced more strongly by the double bond, whereas the amino group is more affected by the structurally proximal 4-CF_3_-substituent. Conversely, the backbone conformational properties and the s-*trans*/s-*cis* energy differences remain nearly non-affected in both cases. Finally, the 3,4-double bond was found to increase the barrier of the amide rotation presumably due to the repulsive effect between the amide oxygen and the double bond in the *syn*/*exo* transition state. Thus 3,4-dehydroproline can be considered as a potential structural ‘freezer’ for polypeptide structures.

## Supporting Information

The crystal structures are deposited in Cambridge Structural Database under the following IDs: **5**- CCDC1443104, **6**- CCDC1443105, **8**- CCDC1443103, **9**- CCDC1443102. The crystal structure of **7** have already been discussed in [35] and the deposit number was CCDC1042476. The structure files can be retrieved free of charge at http://www.ccdc.cam.ac.uk.

File 1 Experimental procedures, values for the amide rotational barriers in different solvents, copies of the NMR spectra and ellipsoid diagrams of the X-ray crystal structures.
