# Supplementary material for: cis–trans-Amide isomerism of the 3,4-dehydroproline residue, the ‘unpuckered’ proline
Source: Beilstein J Org Chem. 2016 Mar 29;12:589–93. doi: 10.3762/bjoc.12.57 (PMC4901939; doi:10.3762/bjoc.12.57)

Supporting Information

for

cis–trans-Amide isomerism of the 3,4-dehydroproline residue, the 'unpuckered' proline

Vladimir Kubyshkin*, Nediljko Budisa*

Address: Institute of Chemistry, Technical University of Berlin, Müller-Breslau-Str., 10, 10623, Berlin, Germany

Email: Vladimir Kubyshkin - kubyshkin@win.tu-berlin.de;

Nediljko Budisa - nediljko.budisa@tu-berlin.de

*Corresponding author

Experimental procedures, values for the amide rotational barriers in different solvents, copies of the NMR spectra and ellipsoid diagrams of the X-ray crystal structures

Index

General experimental information………………………………… S2

Synthesis and characterization of compounds:

- **9**……………………………………………………………… S2
- **3*HCl**…………………………………………………….. S2
- **4*HCl**……………………………………………………... S3
- **6**……………………………………………………………… S3
- **8**……………………………………………………………… S4

Physicochemical parametrization………………………………… S6

Solvent effect on the amide rotation……………………………… S8

Copies of the NMR spectra………………………………………… S10

Ellipsoid plots of the X-ray crystal structures…………………… S31

General experimental information

Starting compounds, reagents and solvents were obtained from commercial sources unless otherwise specified with a reference. Racemic Ac-Pro-OMe was obtained in the procedure analogous to **6** starting from *rac*-Pro methyl ester hydrochloride; and crystals for X-ray diffraction were obtained after long incubation (several months) at 4 °C. The other crystals suitable for X-ray analysis were obtained upon crystallization from dichloromethane.

^{1}H, ^{13}C and ^{19}F NMR spectra are given in δ-scale, and the referencing was achieved using conventional deuterium lock referencing prior the spectra acquisition. The spectra were recorded at either 296 or 298 K. ^{13}C NMR spectra were recorded with proton decoupling during the acquisition. ^{19}F NMR spectra were recorded without decoupling. ^{13}C resonance multiplicities were all singlets unless the substance contained fluorine; in the latter case all multiplicities are specified. The spectral assignment was achieved using ^{1}H NOESY, $^{13}C\{^{1}H\}$ dept45, $^{1}H\{^{13}C\}$ HSQC and $^{1}H^{13}C$ HMBC experiments and pH titration. In the spectral descriptions α-, β-, γ- and δ-letters designate 2-, 3-, 4- and 5-positions in the pyrrolidine/pyrroline rings respectively. Mass-spectra were recorded using electrospray ionization. Optical rotation was measured at 24 °C.

1-(*tert*-Butoxycarbonyl)-4-(trifluoromethyl)-2,5-dihydro-1*H*-pyrrole-2-carboxylic acid (9)

Boc-TfmDhp-OMe [1] (6.19 g, 21.0 mmol) was dissolved in methanol (60 ml) followed by addition of 1 M sodium hydroxide solution (25 ml, 25 mmol). Resulting mixture was stirred at 16 °C for 2 hours. Methanol was removed under reduced pressure (at ≤ 26 °C). Water (150 ml) was added, and aqueous solution was washed with TBME (3 x 100 ml). The aqueous phase was then acidified with 13% hydrogen chloride solution (14 ml) to pH ≈ 1 (pH paper) and extracted with dichloromethane (3 x 100 ml). Dichloromethane fractions were dried over sodium sulfate, filtered and concentrated in vacuum to give **9** (3.17 g, 54% yield) as a yellowish solid, T_m 110-115 °C. A low value of the optical rotation ($[\alpha]_D$ = -9, c = 1.0, MeOH) and a centrosymmetric crystal structure indicate that nearly full racemization of the stereocenter occurred during this step. ^{1}H NMR (MeOD, 700 MHz), two rotamers (5:3), δ: 6.53 (minor) and 6.50 (major) (2m, 1H, β-CH=), 5.14 (m, 1H, α-CH), 4.41-4.34 (m, 2H, δ-CH_2), 1.52 (minor) and 1.47 (major) (2s, 9H, CH_3). ^{13}C NMR (MeOD, 126 MHz), two rotamers, δ: 170.5 and 170.2 (2s, CO_2H), 153.8 and 153.6 (2s, N-C=O), 131.2 (q, J = 35 Hz, γ-C=), 129.7 and 129.5 (2q, J = 5 Hz, β-CH=), 121.1 (q, J = 269 Hz, CF_3), 81.0 and 80.9 (2s, CMe_3), 66.8 and 66.5 (2s, α-CH), 50.6 and 50.3 (2s, δ-CH_2), 27.2 and 27.0 (2s, CH_3). ^{19}F NMR (MeOD, 471 MHz), two rotamers, δ: -67.14 (minor) and -67.16 (major) (CF_3). IR bands (cm^{-1}): 2984, 1742, 1683, 1652. Mass-spectrum: 182.04 $[M+H-Boc]^+$. Anal. Calcd for $C_{11}H_{14}F_3NO_4$: H 5.02, C 46.98; found: H 4.89, C 45.65.

(2*S*,4*S*)-4-(Trifluoromethyl)pyrrolidine-2-carboxylic acid hydrochloride (3*HCl)

Boc-TfmPro [1] (2.43 g; 8.58 mmol) was dissolved in anhydrous dioxane (5 ml) followed by addition of 4 M hydrogen chloride dioxane solution (10 ml, 40 mmol). The resulting mixture was stirred for 3 hours at room temperature. The dioxane was removed under reduced pressure, and the residue was freeze-dried from water. The crude compound was dissolved in water and

filtered with activated charcoal (0.9 g). The filtrate was freeze-dried to give **3***HCl (1.84 g, 98% yield) as a greenish solid, T_m > 130 °C. ^{1}H NMR (D_2O, 700 MHz), δ: 4.29 (t, J = 8.5 Hz, 1H, α-CH), 3.61 (dd, J = 13, 9 Hz, 1H, δ-CH), 3.52 (dd, J = 13, 7 Hz, 1H, δ-CH), 3.35 (m, 1H, γ-CH), 2.66 (dt, J = 14, 9 Hz, 1H, β-CH), 2.22 (dt, J = 14, 8 Hz, 1H, β-CH). ^{13}C NMR (D_2O, 126 MHz), δ: 172.3 (s, CO_2H), 126.0 (q, J = 278 Hz, CF_3), 61.0 (s, α-CH), 44.3 (s, δ-CH_2), 40.8 (q, J = 30 Hz, γ-CH), 28.0 (s, β-CH_2). ^{19}F NMR (D_2O, 659 MHz), δ: -71.2 (d, J = 9 Hz, CF_3). $[\alpha]_D$ = -16 (c = 1.0, MeOH). IR bands (cm^{-1}): 2879, 1734. Mass-spectrum: 184.06 [**3**+H]$^+$. Anal. Calcd for $C_6H_9ClF_3NO_2$: H 4.13, C 32.82; found: H 4.39, C 32.67.

1-Acetyl-4-(trifluoromethyl)-2,5-dihydro-1H-pyrrole-2-carboxylic acid. Compound **4***HCl (29 mg, 0.13 mmol) was mixed with acetic anhydride (200 μl) in dichloromethane (2 ml). The resulting turbid mixture was vigorously stirred for the next 16 hours at room temperature to give a clear solution. Dichloromethane was removed using a nitrogen flow, water (1.5 ml) was added, and the mixture was freeze-dried. The residue was freeze dried from water to give Ac-TfmDhp (30 mg, quant.) as a white powder. The NMR data is given for the salt form (phosphate buffer, pH 7). ^{1}H NMR (buffer, D_2O, 700 MHz), two rotamers (1:1), δ: 6.48 (s-*cis*) and 6.46 (s-*trans*) (2sept., J = 2 Hz, 1H, β-CH=), 5.15 (s-*cis*, sext., J = 2.5 Hz) and 5.03 (s-*trans*, sept., J = 3 Hz) (1H, α-CH), 4.57 (s-*trans*, m), 4.44 and 4.35 (2dm, J = 16 Hz) (2H, δ-CH_2), 2.07 (s-*trans*) and 1.95 (s-*cis*) (2s, 3H, CH_3). ^{13}C NMR (buffer, D_2O, 176 MHz), two rotamers, δ: 174.6 (s-*trans*) and 174.4 (s-*cis*) (2s, CO_2^-), 173.1 (s-*cis*) and 172.3 (s-*trans*) (2s, N-C=O), 131.2 (m, β-CH=), 129.0 (m, γ-C=), 121.1 and 120.9 (2q, J = 270 Hz, CF_3), 70.8 (s-*cis*) and 69.6 (s-*trans*) (2s, α-CH), 52.0 (s-*trans*) and 50.9 (s-*cis*) (2s, δ-CH_2), 21.0 (s-*trans*) and 20.6 (s-*cis*) (2s, CH_3). ^{19}F NMR (buffer, D_2O, 659 MHz), two rotamers (1:1), δ: -65.29 (s-*trans*) and -65.31 (s-*cis*) (CF_3). IR bands (cm^{-1}): 2930, 1738, 1607. Mass-spectrum: 224.05 [M+H]$^+$. Anal. Calcd for $C_8H_8F_3NO_3$: H 3.61, C 43.06; found: H 3.90, C 43.01.

4-(Trifluoromethyl)-2,5-dihydro-1*H*-pyrrole-2-carboxylic acid hydrochloride (4*HCl)

Compound **9** (47 mg, 0.17 mmol) was dissolved in 4 M hydrogen chloride solution in dioxane (1 ml, 4 mmol). The mixture was stirred for 3 hours, the dioxane was removed under reduced pressure, and the residue was dissolved in water and freeze-dried. Compound **4***HCl (34 mg, 94 % yield) was obtained as a brown solid. ^{1}H NMR (D_2O, 700 MHz), δ: 6.64 (sept., J = 2 Hz, 1H, β-CH=), 5.21 (sept. J = 2.7 Hz, 1H, α-CH), 4.42 and 4.34 (2dm, J = 15 Hz, 2H, δ-CH_2). ^{13}C NMR (D_2O, 126 MHz), δ: 168.9 (s, CO_2H), 130.3 (q, J = 5 Hz, β-CH=), 128.3 (q, J = 37 Hz, γ-C=), 120.2 (q, J = 270 Hz, CF_3), 68.0 (s, α-CH), 49.6 (s, δ-CH_2). ^{19}F NMR (D_2O, 471 MHz), δ: -65.0 (s, CF_3). IR bands (cm^{-1}): 2848, 1746, 1680. Mass-spectrum: 182.04 [**4**+H]$^+$. Anal. Calcd for $C_8H_8F_3NO_3$: H 3.61, C 43.06; found: H 3.90, C 42.80. Anal. Calcd for $C_6H_7ClF_3NO_2$: H 3.24, C 33.12; found: H 3.60, C 32.88.

Methyl (*S*)-1-acetyl-2,5-dihydro-1*H*-pyrrole-2-carboxylate (6)

Methyl 3,4-dehydroprolinate hydrochloride (124 mg, 0.75 mmol) was mixed with acetic anhydride (200 μl) in dichloromethane (3 ml) at room temperature for 20 min. Dichloromethane was removed under reduced pressure, the residue was dissolved in a minimal amount of water and freeze-dried. The crude compound was purified on a silica gel column (ethyl acetate–

methanol 19:1 as eluent) to give **6** (71 mg, 55% yield) as a colorless solid, T_m 70-75 °C. ^{1}H NMR (D_2O, 700 MHz), two rotamers, δ : 6.04 (m, 1H, γ-CH=), 5.81 (minor) and 5.78 (major) (2m, 1H, β-CH=), 5.32 (minor) and 5.09 (major) (two m, 1H, α-CH), 4.37 (major, dm, J = 15 Hz), 4.33 (major, dm, J = 15 Hz), 4.24 (minor, dm, J = 16 Hz) and 4.12 (minor, dm, J = 16 Hz) (2H, δ-CH_2), 3.73 (minor) and 3.69 (major) (2s, 3H, CH_3O), 2.06 (major) and 1.94 (minor) (2s, 3H, CH_3C=O). ^{13}C NMR (D_2O, 126 MHz), two rotamers, δ: 173.3 (minor N-C=O), 172.8 (major, N-C=O), 172.6 (major CO_2Me), 172.3 (minor CO_2Me), 129.5 (major γ-CH=), 128.9 (minor γ-CH=), 124.0 (minor β-CH=), 123. 6 (major β-CH=), 67.3 (minor α-CH), 66.2 (major, α-CH), 54.7 (major δ-CH_2), 53.6 (minor δ-CH_2), 53.4 (minor CH_3O), 53.2 (major CH_3O), 20.85 (minor *C*H_3C=O), 20.83 (major *C*H_3C=O). $[\alpha]_D$ = -337 (c = 1.0, $CHCl_3$). IR bands (cm^{-1}): 3084, 2952, 1744, 1637, 1618. Mass-spectrum: 170.08 $[M+1]^+$. Anal. Calcd for $C_8H_{11}NO_3$: H 6.55, C 56.80; found: H 6.50, C 56.59.

(S)-1-Acetyl-2,5-dihydro-1H-pyrrole-2-carboxylic acid. Compound **6** (35 mg, 0.21 mmol) was dissolved in water (2 ml) followed by addition of 1 M sodium hydroxide solution (215 μl). The mixture was stirred at the room temperature for 1.5 hours. The aqueous solution was then washed with dichloromethane (1 x 2 ml), and freeze-dried. 1 M hydrogen chloride aqueous solution (300 μl) and water (2 ml) were added, and resulting solution was freeze-dried. The pK_a values of the target compound were measured without isolation of the material from the remaining sodium chloride. The NMR data is given for the salt form (phosphate buffer, pH 7). ^{1}H NMR (buffer, D_2O, 700 MHz), two rotamers (1:1), δ: 5.94 (m, 1H, γ-CH=), 5.81 (m, 1H, β-CH=), 4.98 and 4.87 (2sext., J = 2.2 Hz, 1H, α-CH), 4.36 (dm, J = 15 Hz), 4.32 (dq, J = 15, 2.1 Hz), 4.22 (dq, J = 16, 2 Hz) and 4.16 (ddt, J = 16, 5 and 2 Hz) (2H, δ-CH_2), 2.06 and 1.94 (2s, 3H, CH_3). ^{13}C NMR (buffer, D_2O, 126 MHz), two rotamers, δ: 177.1 (s-*trans*) and 177.0 (s-*cis*) (CO_2^-), 173.0 (s-*cis*) and 172.2 (s-*trans*) (N-C=O), 127.1 and 126.9 (γ-CH=), 126.4 and 126.3 (β-CH=), 70.7 (s-*cis*) and 69.2 (s-*trans*) (α-CH), 54.9 (s-*trans*) and 53.6 (s-*cis*) (δ-CH_2), 21.1 (s-*trans*) and 20.9 (s-*cis*) (CH_3).

Methyl (*S*)-1-acetyl-4-(trifluoromethyl)-2,5-dihydro-1*H*-pyrrole-2-carboxylate (8)

Methyl (S)-4-(trifluoromethyl)-2,5-dihydro-1H-pyrrole-2-carboxylate. Boc-TfmDhp-OMe [1] (110 mg, 0.37 mmol) was dissolved in 4 M hydrogen chloride solution in dioxane (1.5 ml, 6.0 mmol). (*NB*: lower acid concentrations (i.e. 2 M HCl/dioxane) result in only partial removal of the Boc-group, due to the low basicity of the amine functionality. See Table 1) The resulting mixture was stirred for 10 hours at room temperature, dioxane was removed under reduced pressure, water was added and the aqueous solution was freeze-dried to give HCl*TfmDhp-OMe as a white powder. ^{1}H NMR (D_2O, 700 MHz), δ: 6.70 (sept., J = 2 Hz, β-CH=), 5.51 (sept. J = 2.7 Hz, 1H, α-CH), 4.45 and 4.39 (2dm, J = 15 Hz, 2H, δ-CH_2), 3.84 (s, 3H, CH_3O). ^{19}F NMR (D_2O, 471 MHz), δ: -65.2 (s, CF_3).

This salt was then mixed with dichloromethane (4 ml), and acetic anhydride (200 μl) was added to the turbid mixture. The mixture was stirred at room temperature for 1.5 hours, additional acetic anhydride (200 μl) was added, and stirring was continued for the next 1.5 hours. When the resulting solution was clear, the dichloromethane was removed under reduced pressure, water was added in order to quench remaining anhydride, and aqueous solution was freeze-

dried. Compound **8** (84 mg, 95% yield) was obtained as a yellowish crystalline solid, T_m 65-70 °C. ^{1}H NMR (D_2O, 700 MHz), two rotamers, δ: 6.54 (minor) and 6.49 (major) (2m, 1H, β-CH=), 5.56 (minor) and 5.29 (major) (2m, 1H, α-CH), 4.61 (m, major) and 4.48 and 4.33 (2dm, J = 16 Hz, minor) (2H, δ-CH_2), 3.77 (minor) and 3.72 (major) (2s, CH_3O), 2.09 (major) and 1.97 (monor) (2s, CCH_3). ^{13}C NMR (D_2O, 176 MHz), two rotamers, δ: 173.3 (minor) and 172.9 (major) (2s, N-C=O), 170.5 (major) and 170.3 (minor) (2s, CO_2Me), 131.4 (major) and 130.8 (minor) (2q, J = 36 Hz, γ-C=), 128.9 (minor) and 128.4 (major) (2q, J = 5 Hz, β-CH=), 120.8 (minor) and 120.5 (major) (2q, J = 269 Hz, CF_3), 67.2 (minor) and 66.4 (major) (2s, α-CH), 53.7 (minor) and 53.4 (major) (2s, CH_3O), 51.8 (major) and 50.9 (minor) (2s, δ-CH_2), 20.7 (major) and 20.6 (minor) (2s, *C*H_3C=O). ^{19}F NMR (D_2O, 659 MHz), two rotamers, δ: -65.6 (minor) and -65.7 (major) (2s, CF_3). $[\alpha]_D$ = -238 (c = 1.0, $CHCl_3$). IR bands (cm^{-1}): 3083, 2961, 1750, 1646. Mass-spectrum: 238.07 $[M+1]^+$. Anal. Calcd for $C_9H_{10}F_3NO_3$: H 4.25, C 45.58; found: H 4.40, C 45.32.

Physicochemical parametrization

All pK_a parameters were determined at 298 K by NMR of buffered solutions which contained analyte and potassium phosphate buffer (3–10 mM) or/and citrate buffer. These solutions were titrated with hydrogen chloride and potassium hydroxide in a suitable pH range. 500 µl aliquots were taken at different pH values, and 55 µl deuterium oxide was added for lock and shimming. Samples also contained 0.1 mM of sodium 3-(trimethylsilyl)-1-propanesulfonate for ^{1}H referencing. The ^{1}H NMR spectra were collected with bulk water suppression using W5 pulse tray. ^{19}F NMR spectra were collected in single-pulse experiments. Chemical shifts were plotted against pH, fitted according to Boltzmann fit. 1st order derivative of a fit curve indicated the pK_a value as the extremum point.

Rotational rates were determined in solutions of Ac-Xaa-OMe compounds by ^{1}H 2D cross-relaxation experiments (NOESY-EXSY). The spectra were recorded at 310 K according to the methanol temperature calibration [2]. Mixing time was 1 and 2 s for exchange and 5 ms for referencing; the recycling delay was chosen as ≥ $3 \cdot T_1$ for the analyzed resonances. Time domain was inset such that the resolution was 5–2 Hz in the direct and 20–10 Hz in the indirect dimensions respectively. The resolution of the indirect dimension was zero-filled to reach the direct dimension resolution and the time domain spectra were windowed (squared sine bell function) and Fourier transformed. Frequency domain spectra were baseline corrected and integrated. The integrals were analyzed using EXSYCalc® freeware to give corresponding rotational rates (k). Activation energies were derived from these values using Eyring equation as following:

$$E^{\neq} = -(\ln(k/T) - 23.76) \cdot T \cdot R$$

, where T – absolute temperature, R – gas constant, and $\ln(k_B/h) = 23.76$ (k_B – Boltzmann constant, h – Planck constant). The pK_a values and the rotational rates for Pro and its 4-substituted derivatives were collected from the literature [1,3,4].

The equilibrium s-*trans*/s-*cis* population ratios were determined by ^{1}H NMR of the deuterium oxide solutions at 700 MHz proton frequency and 296 K. The spectra were recorded in one scan in order to ensure complete relaxation of the nuclei. This precaution was especially valid for Dhp derivatives, where several resonances exhibited drastically high relaxation times, producing distorted integrals in multiscan experiments with standard relaxation delays.

References

1. Kubyshkin, V.; Afonin, S.; Kara, S.; Budisa, N.; Mykhailiuk, P. K.; Ulrich, A. S. *Org. Biomol. Chem.* **2015**, *13*, 3171-3181
2. Van Geet, A. L. *Anal. Chem.* **1970**, *42*, 679-680

3. Renner, C.; Alefelder, S.; Bae, J. H.; Budisa, N.; Huber, R.; Moroder, L. *Angew. Chem., Int. Ed.* **2001,** *40,* 923–925. doi:10.1002/1521-3773(20010302)40:5<923::AID-ANIE923>3.0.CO;2-#
4. Doerfel, L.; Wohlgemuth, I.; Kubyshkin, V.; Starosta, A. L.; Wilson, D. N.; Budisa, N.; Rodnina, M. V. *J. Am. Chem. Soc.* **2015,** *137,* 12997–13006. doi:10.1021/jacs.5b07427

Solvent effect on the amide rotation

The amide rotation barrier have been also determined for AcXaaOMe in other organic solvents: MeOD, DMSO and $CDCl_3$. Resulting values were correlated to the solvent's Reichardt–Dimroth paramether, which reflects the ability of solvents to separate charges. The charge separation should affect the amide ground state energy according to the following scheme:

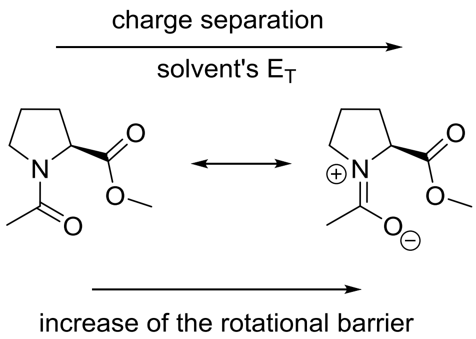

The amide rotation activation energies were determined at 310 K. *E*a mean designates the mean value of the *s-cis*-to-*s-trans* and *s-trans*-to-*s-cis* isomerization barriers for each solvent/compound pair. The resulting correlation indicates similarities in the charge separation in the ground states, thus the activation energy offset in Dhp and tfmDhp is more likely to originate from the transition state effects as claimed in the main text.

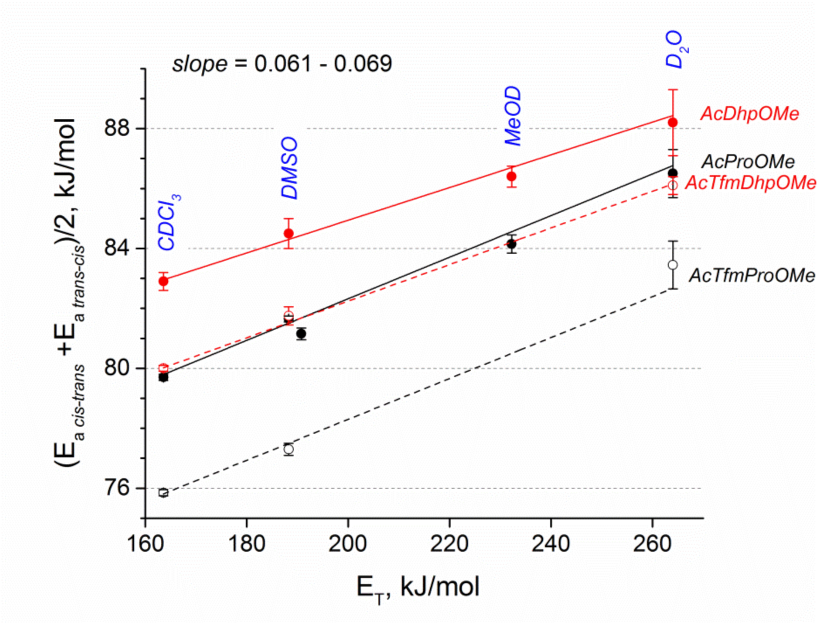

Found activation energies (310 K):

E_T, kJ/mol	solvent	E_a, kJ/mol							
		AcProOMe, **5**		AcTfmProOMe, **7**		AcDhpOMe, **6**		AcTfmDhpOMe, **8**	
		c→t	t→c	c→t	t→c	c→t	t→c	c→t	t→c
264.01	D_2O	84.5±0.8	88.5±0.9	81.4±0.2	84.9±0.3	86.1±1.4	90.3±0.7	84.0±0.4	88.2±0.2
232.21	MeOD	82.6±0.2	85.7±0.4	–	–	84.9±0.4	87.9±0.3	–	–
188.28	DMSO	$80.1\pm0.0_4$	83.2±0.1	75.9±0.2	78.7±0.1	83.3±0.4	85.7±0.5	80.1±0.4	83.4±0.2
163.59	$CDCl_3$	$78.0\pm0.0_5$	81.4±0.1	$74.4\pm0.0_3$	$77.3\pm0.0_3$	82.0±0.2	83.8±0.4	$78.5\pm0.0_3$	$81.5\pm0.0_5$

^{1}H NMR spectrum of compound **9** (Boc-TfmDhp) in MeOD

Current Data Parameters
NAME wova_JT_TfmDhp
EXPNO 131
PROCNO 1

F2 - Acquisition Parameters
Date_ 20151210
Time 10.47
INSTRUM spect
PROBHD 5 mm PATXI 1H/
PULPROG zg
TD 65536
SOLVENT MeOD
NS 1
DS 0
SWH 8333.333 Hz
FIDRES 0.127157 Hz
AQ 3.9321599 sec
RG 41.2
DW 60.000 usec
DE 10.00 usec
TE 299.6 K
D1 2.00000000 sec
TD0 1

======== CHANNEL f1 ========
SFO1 700.1732898 MHz
NUC1 1H
P1 11.50 usec
PLW1 15.00000000 W

F2 - Processing parameters
SI 32768
SF 700.1700000 MHz
WDW EM
SSB 0
LB 0.30 Hz
GB 0
PC 10.00

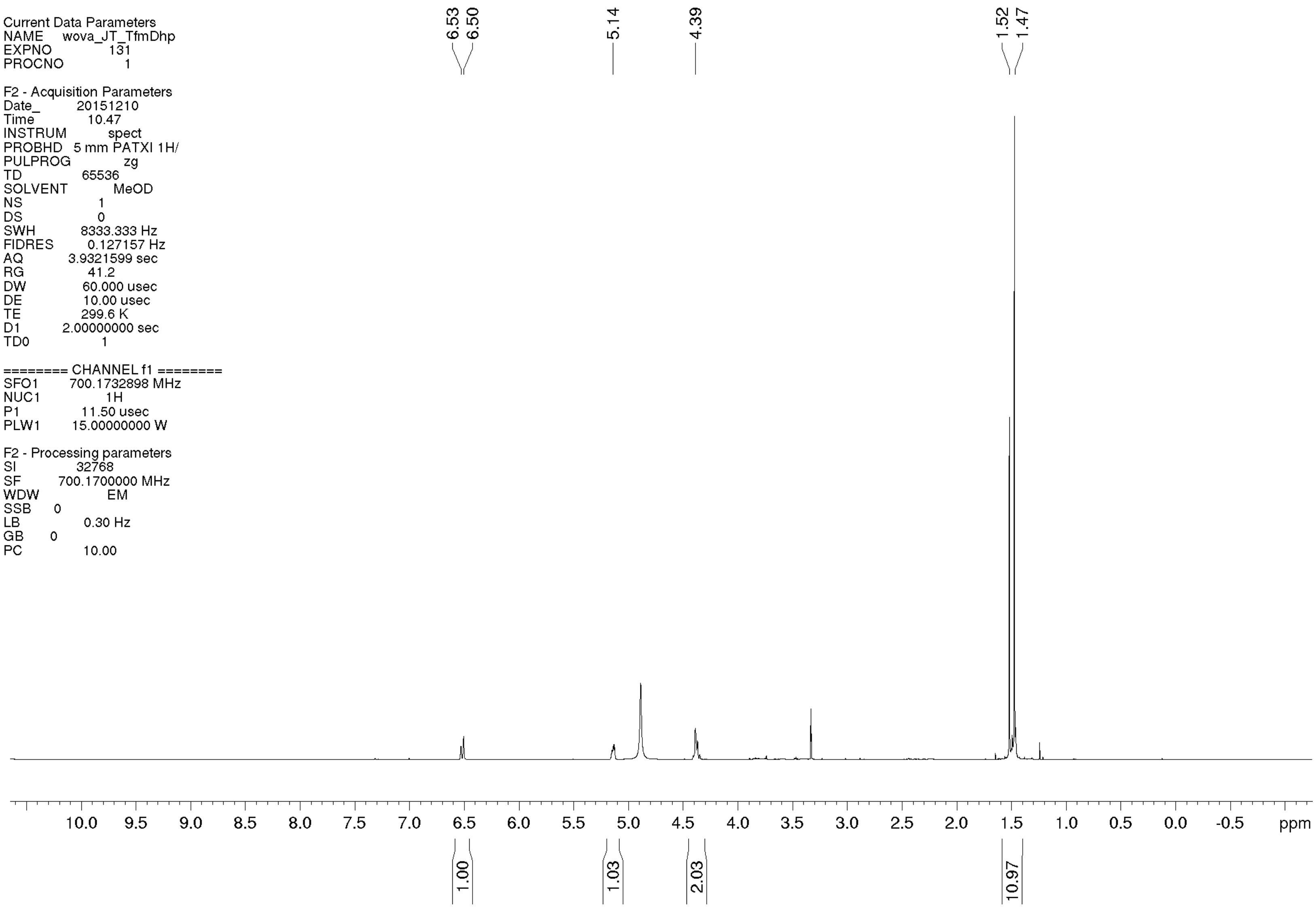

$^{13}C\{^{1}H\}$ NMR spectrum of compound **9** (Boc-TfmDhp) in MeOD

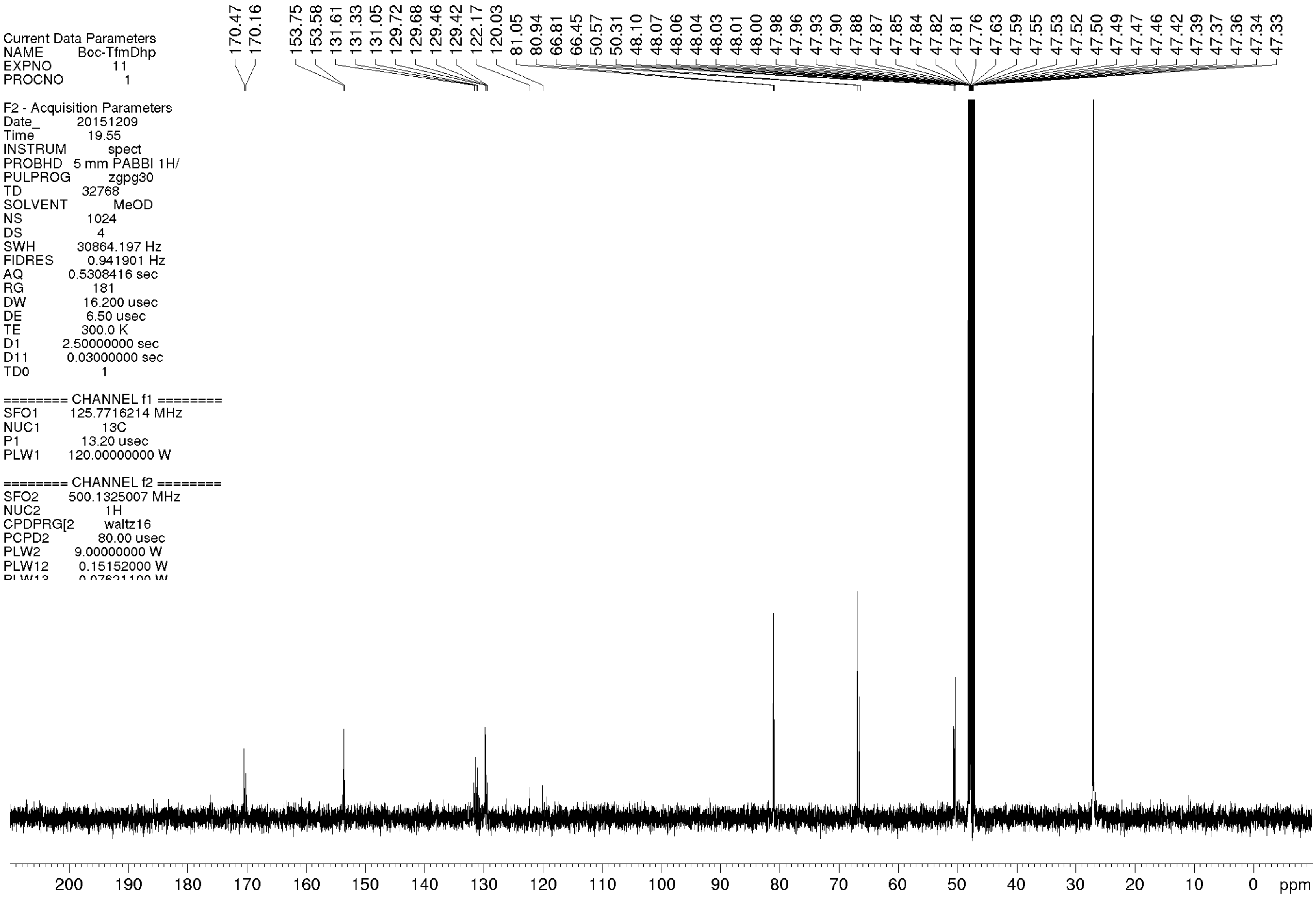

^{19}F NMR spectrum of compound **9** (Boc-TfmDhp) in MeOD

Current Data Parameters
NAME wova_JT_TfmDhp
EXPNO 171
PROCNO 1

F2 - Acquisition Parameters
Date_ 20151210
Time 10.37
INSTRUM spect
PROBHD 5 mm PABBO BB/
PULPROG zg
TD 65536
SOLVENT MeOD
NS 1
DS 0
SWH 33333.332 Hz
FIDRES 0.508626 Hz
AQ 0.9830400 sec
RG 2050
DW 15.000 usec
DE 80.00 usec
TE 298.0 K
D1 2.50000000 sec
TD0 1

======== CHANNEL f1 ========
SFO1 470.6743524 MHz
NUC1 19F
P1 14.75 usec
PLW1 42.00000000 W

F2 - Processing parameters
SI 32768
SF 470.7052900 MHz
WDW EM
SSB 0
LB 0.30 Hz
GB 0
PC 1.00

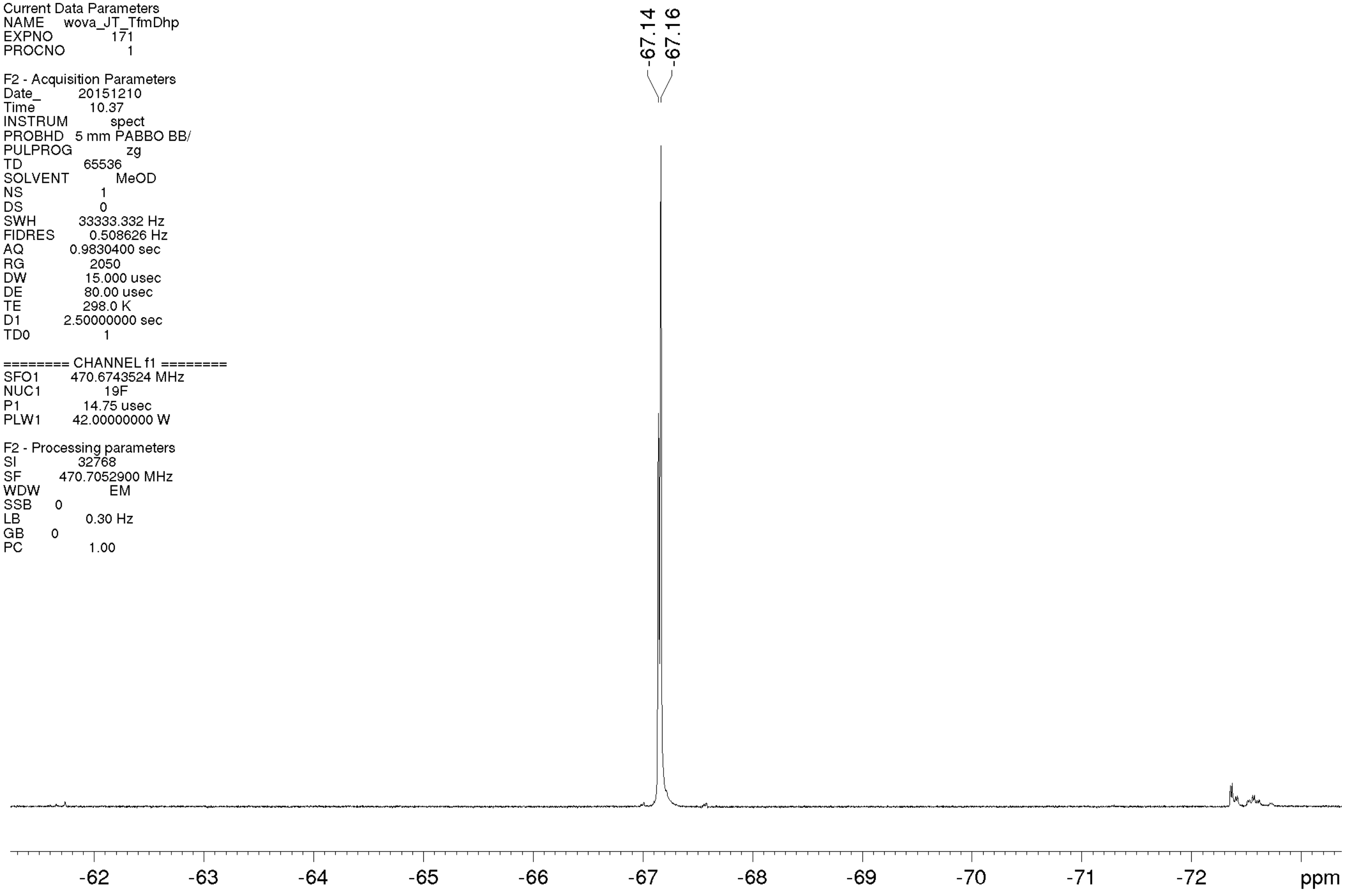

^{1}H NMR spectrum of compound **3***HCl (HCl*TfmPro) in D_2O

Current Data Parameters
NAME wova_TfmPro
EXPNO 83
PROCNO 1

F2 - Acquisition Parameters
Date_ 20141104
Time 17.00
INSTRUM spect
PROBHD 5 mm PATXI 1H/
PULPROG zg
TD 102400
SOLVENT D2O
NS 8
DS 0
SWH 10000.000 Hz
FIDRES 0.097656 Hz
AQ 5.1199999 sec
RG 45.24
DW 50.000 usec
DE 6.50 usec
TE 298.0 K
D1 2.00000000 sec
TD0 1

======== CHANNEL f1 ========
SFO1 700.1732874 MHz
NUC1 1H
P1 12.35 usec
PLW1 15.00000000 W

F2 - Processing parameters
SI 32768
SF 700.1700000 MHz
WDW EM
SSB 0
LB 0.20 Hz
GB 0
PC 10.00

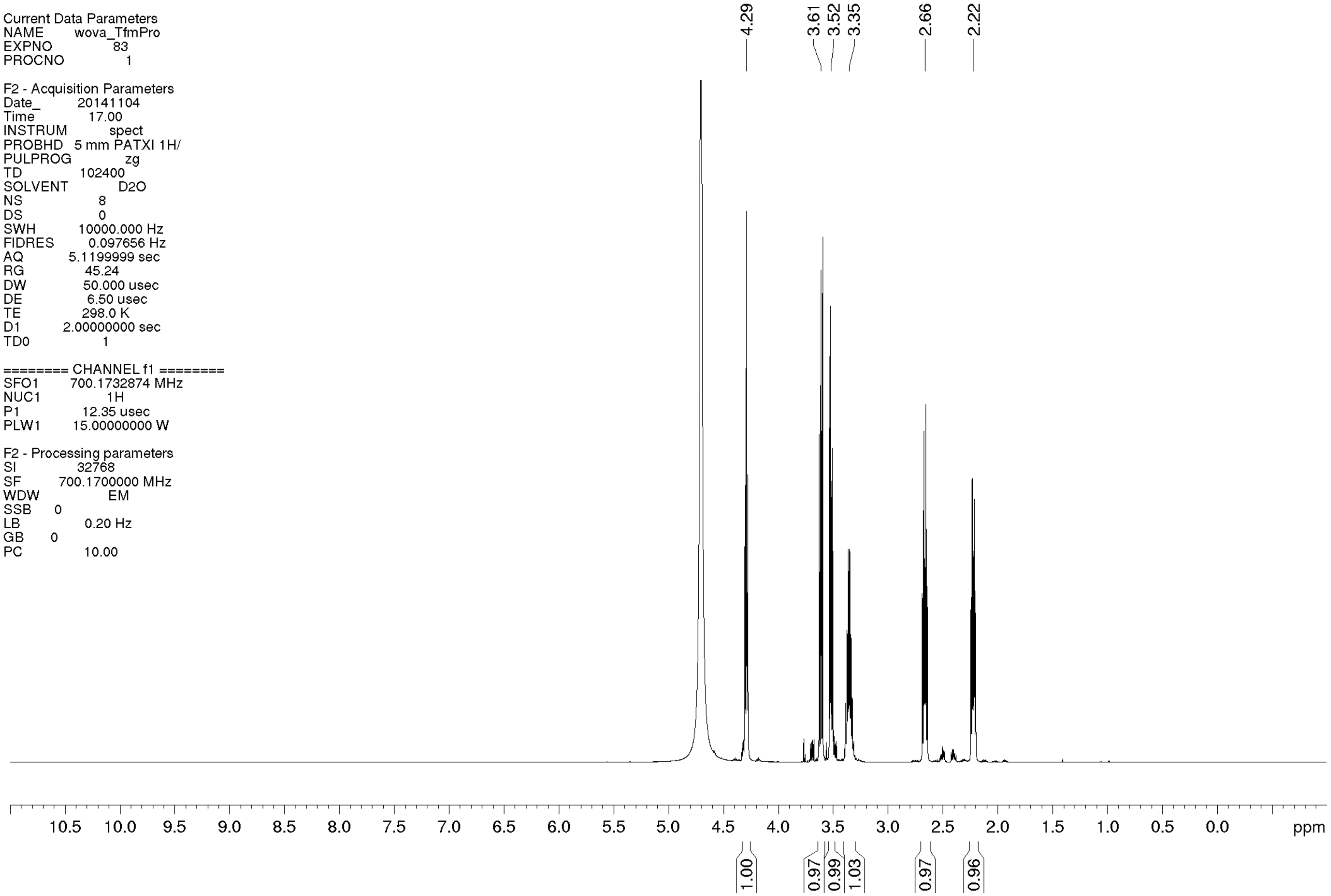

$^{13}C\{^{1}H\}$ NMR spectrum of compound **3***HCl (HCl*TfmPro) in D_2O

Current Data Parameters
NAME wova_TfmPro
EXPNO 85
PROCNO 1

F2 - Acquisition Parameters
Date_ 20141104
Time 17.10
INSTRUM spect
PROBHD 5 mm PATXI 1H/
PULPROG zgig30
TD 65536
SOLVENT D2O
NS 2048
DS 4
SWH 40760.871 Hz
FIDRES 0.621962 Hz
AQ 0.8039083 sec
RG 2050
DW 12.267 usec
DE 6.50 usec
TE 298.1 K
D1 1.50000000 sec
D11 0.03000000 sec
TD0 256

======== CHANNEL f1 ========
SFO1 176.0763711 MHz
NUC1 13C
P1 12.00 usec
PLW1 250.00000000 W

======== CHANNEL f2 ========
SFO2 700.1728007 MHz
NUC2 1H
CPDPRG[2 waltz16
PCPD2 65.00 usec
PLW2 15.00000000 W
PLW12 0.35148999 W

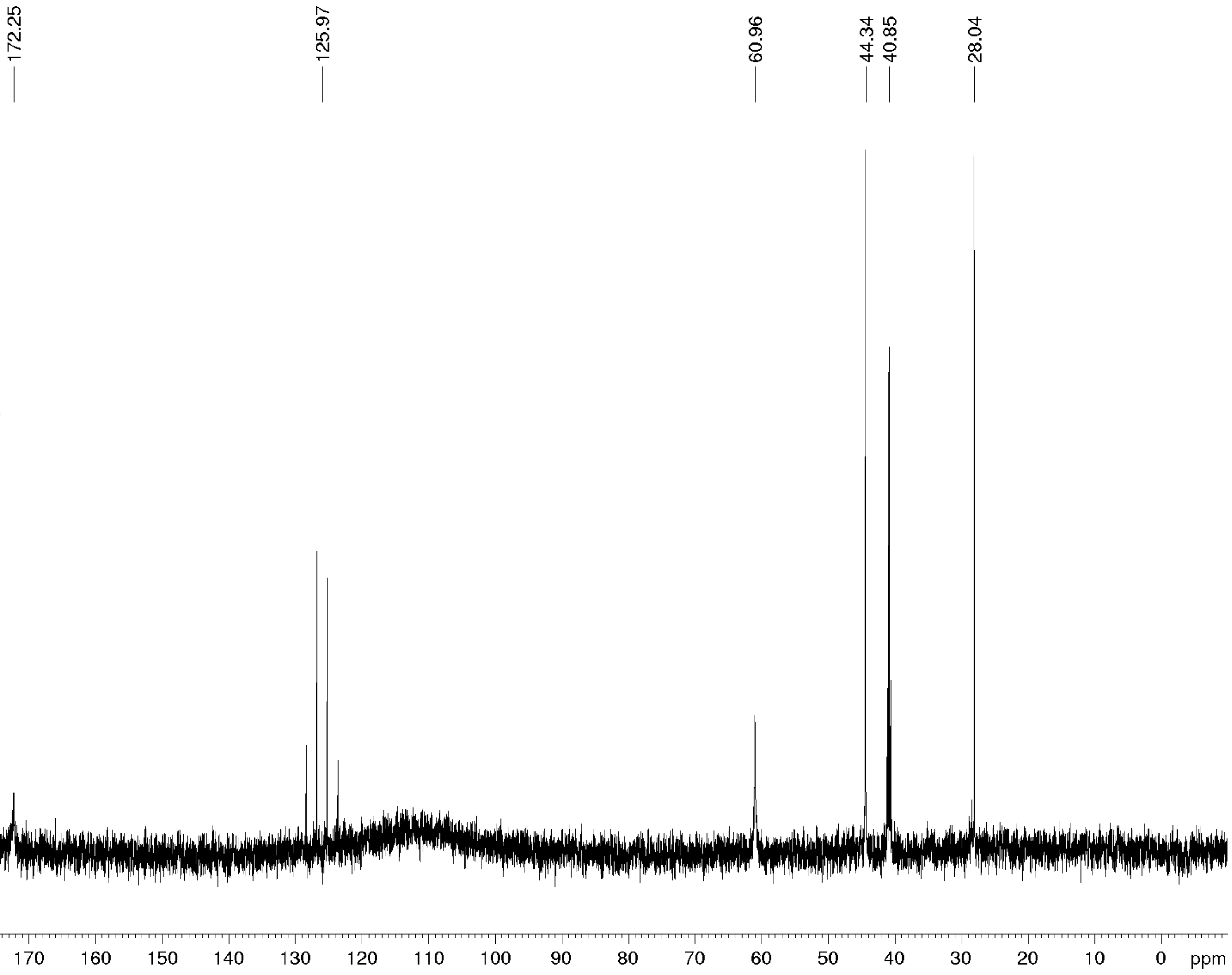

S14

^{19}F NMR spectrum of compound **3***HCl (HCl*TfmPro) in D_2O

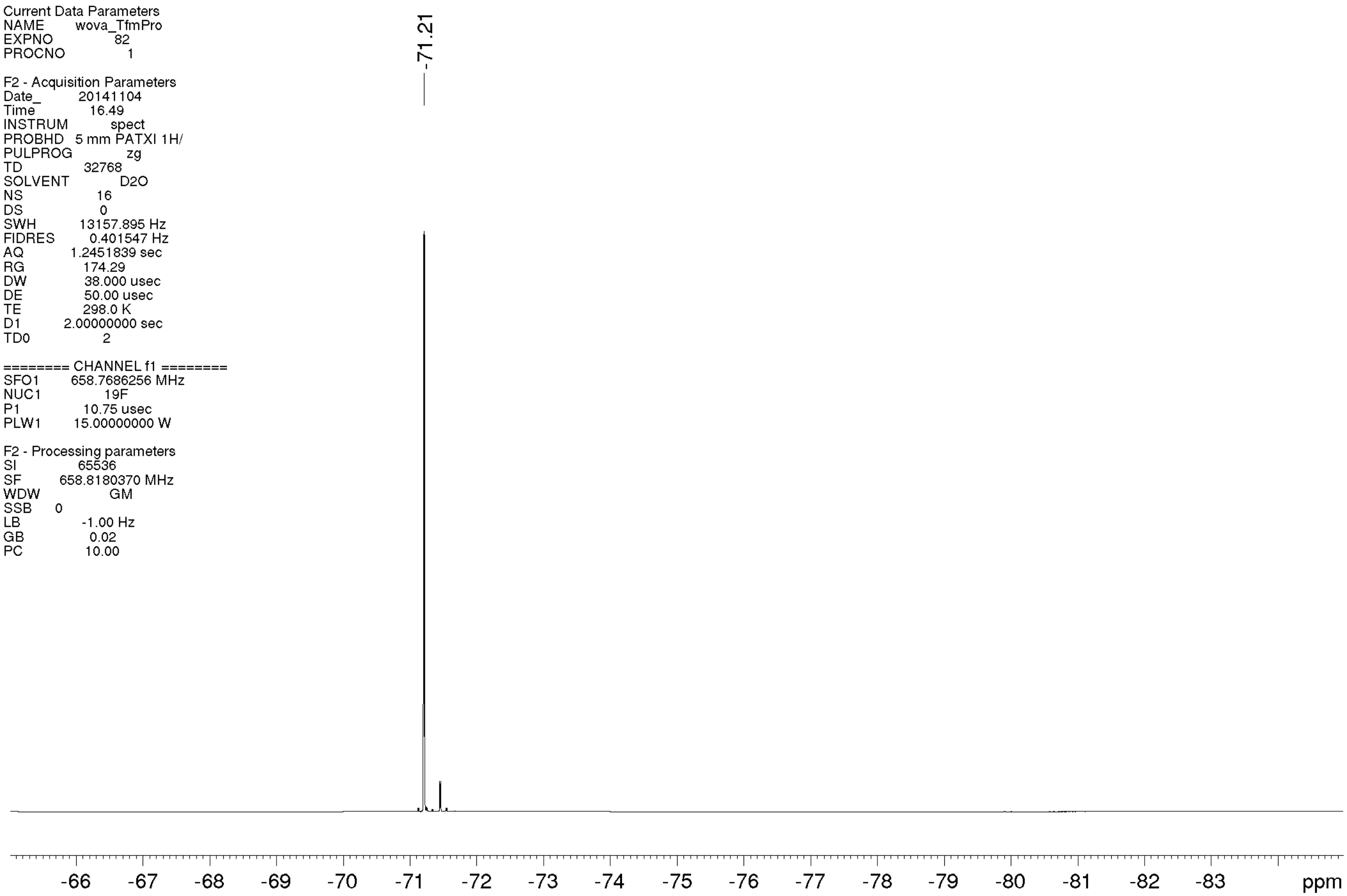

^{1}H NMR spectrum of compound **4***HCl (HCl*TfmDhp) in D_2O

Current Data Parameters
NAME wova_JT_TfmDhp
EXPNO 120
PROCNO 1

F2 - Acquisition Parameters
Date_ 20151210
Time 10.27
INSTRUM spect
PROBHD 5 mm PATXI 1H/
PULPROG zg
TD 65536
SOLVENT D2O
NS 1
DS 0
SWH 8333.333 Hz
FIDRES 0.127157 Hz
AQ 3.9321599 sec
RG 7.99
DW 60.000 usec
DE 10.00 usec
TE 299.6 K
D1 2.00000000 sec
TD0 1

======== CHANNEL f1 ========
SFO1 700.1732898 MHz
NUC1 1H
P1 12.25 usec
PLW1 15.00000000 W

F2 - Processing parameters
SI 32768
SF 700.1700000 MHz
WDW EM
SSB 0
LB 0.30 Hz
GB 0
PC 10.00

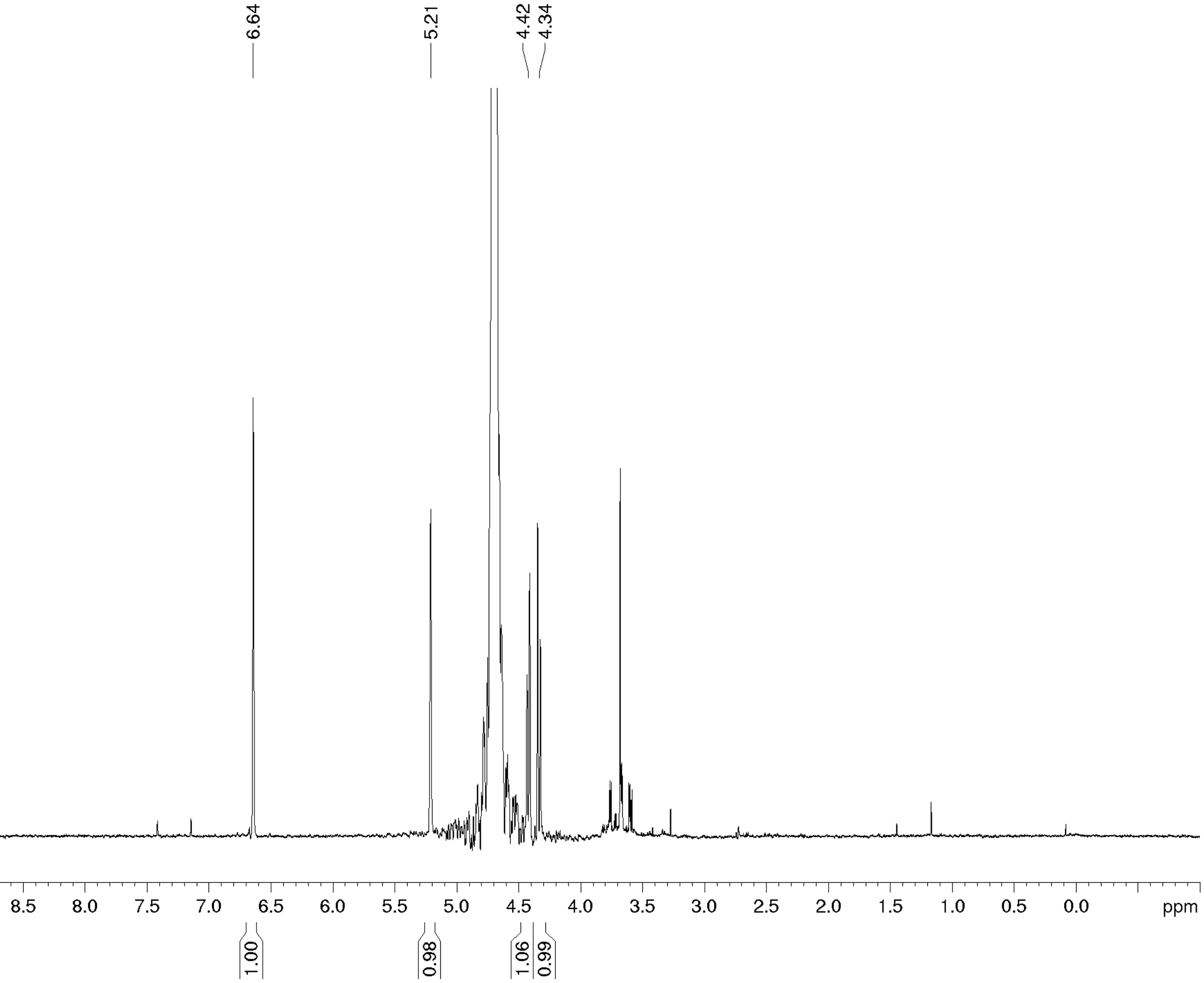

$^{13}C\{^{1}H\}$ NMR spectrum of compound **4***HCl (HCl*TfmDhp) in D_2O

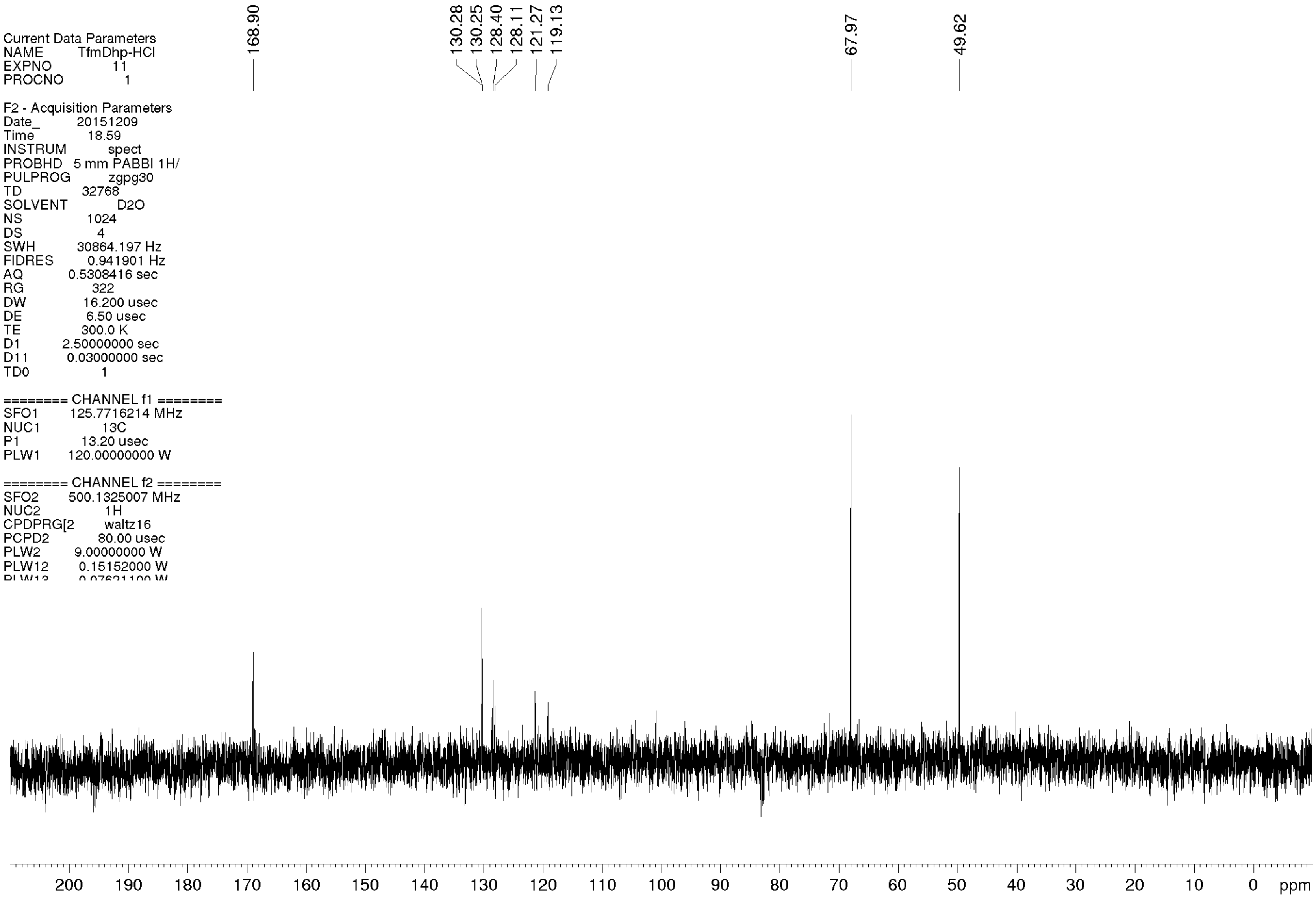

^{19}F NMR spectrum of compound **4***HCl (HCl*TfmDhp) in D_2O

Current Data Parameters
NAME wova_JT_TfmDhp
EXPNO 181
PROCNO 1

F2 - Acquisition Parameters
Date_ 20151210
Time 10.45
INSTRUM spect
PROBHD 5 mm PABBO BB/
PULPROG zg
TD 65536
SOLVENT D2O
NS 1
DS 0
SWH 33333.332 Hz
FIDRES 0.508626 Hz
AQ 0.9830400 sec
RG 2050
DW 15.000 usec
DE 80.00 usec
TE 298.0 K
D1 2.50000000 sec
TD0 1

======== CHANNEL f1 ========
SFO1 470.6743524 MHz
NUC1 19F
P1 14.75 usec
PLW1 42.00000000 W

F2 - Processing parameters
SI 32768
SF 470.7052900 MHz
WDW EM
SSB 0
LB 0.30 Hz
GB 0
PC 1.00

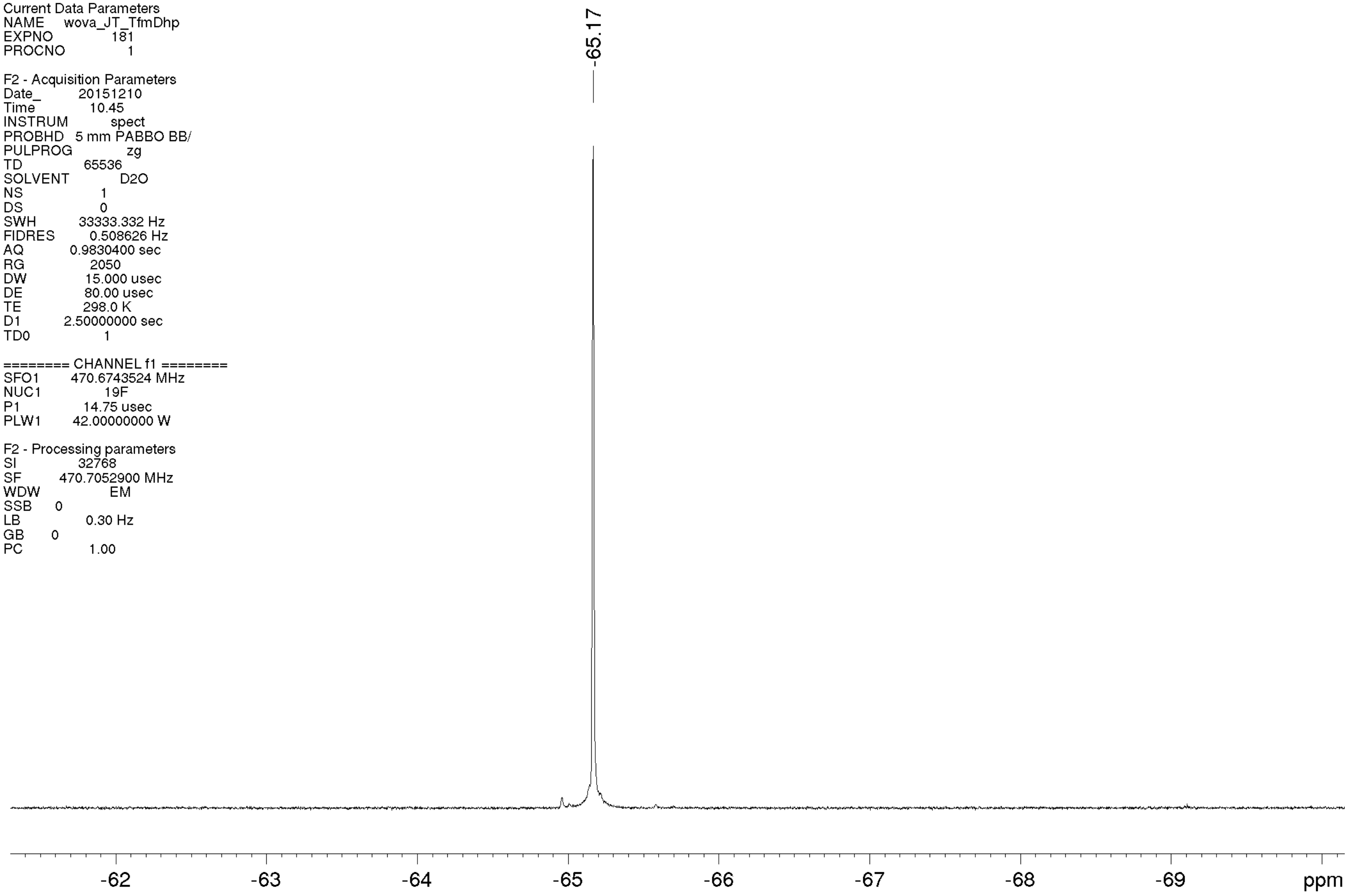

^1H NMR spectrum of Ac-TfmDhp-O^- in D_2O (buffer, pH 7)

Current Data Parameters
NAME wova_JT_TfmDhp
EXPNO 210
PROCNO 1

F2 - Acquisition Parameters
Date_ 20151213
Time 2.49
INSTRUM spect
PROBHD 5 mm PATXI 1H/
PULPROG zg
TD 65536
SOLVENT D2O
NS 1
DS 0
SWH 8333.333 Hz
FIDRES 0.127157 Hz
AQ 3.9321599 sec
RG 88.07
DW 60.000 usec
DE 10.00 usec
TE 299.6 K
D1 2.00000000 sec
TD0 1

======== CHANNEL f1 ========
SFO1 700.1732898 MHz
NUC1 1H
P1 12.75 usec
PLW1 15.00000000 W

F2 - Processing parameters
SI 32768
SF 700.1700000 MHz
WDW GM
SSB 0
LB -0.50 Hz
GB 0.1
PC 10.00

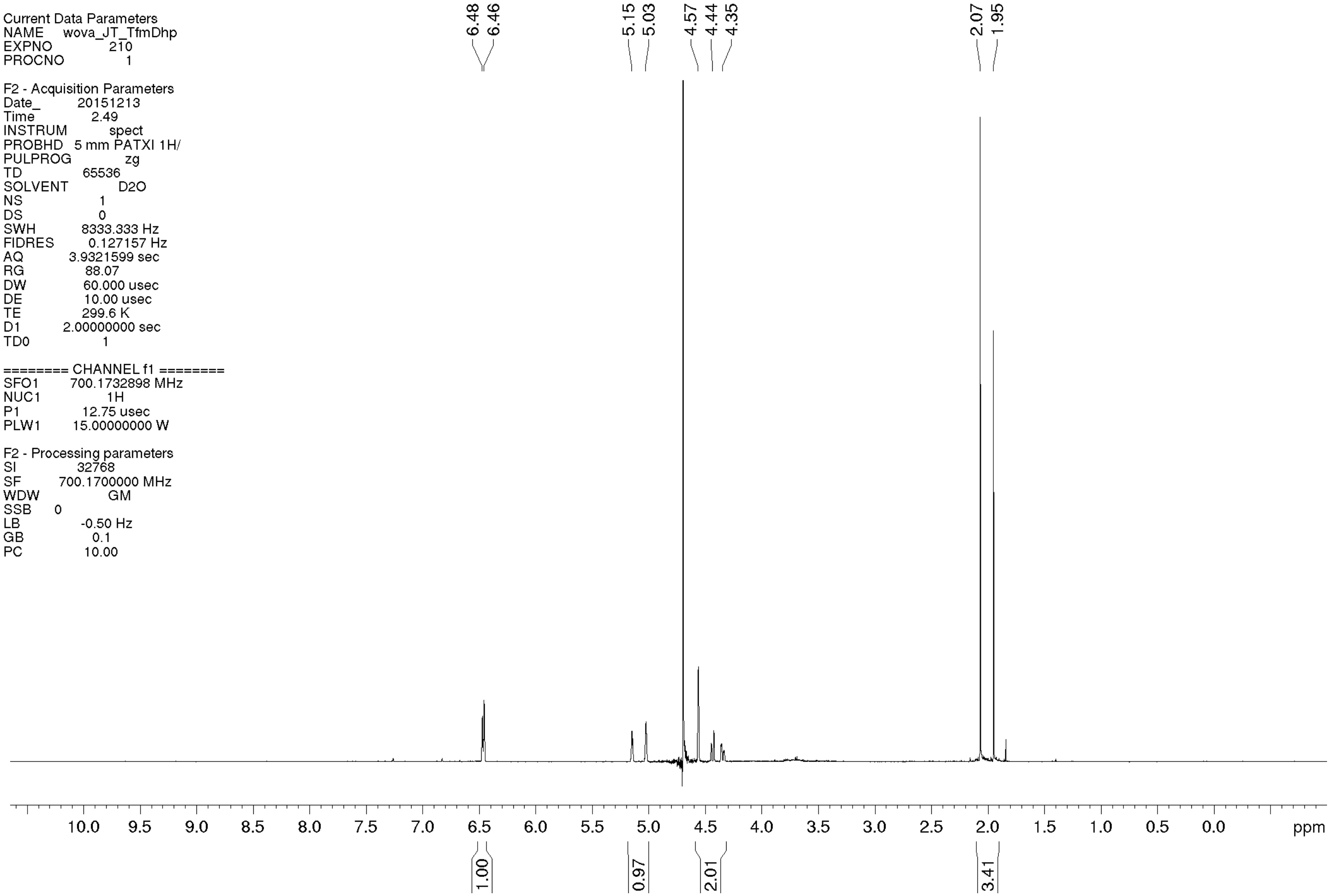

$^{13}C\{^1H\}$ NMR spectrum of Ac-TfmDhp-O$^-$ in D_2O (buffer, pH 7)

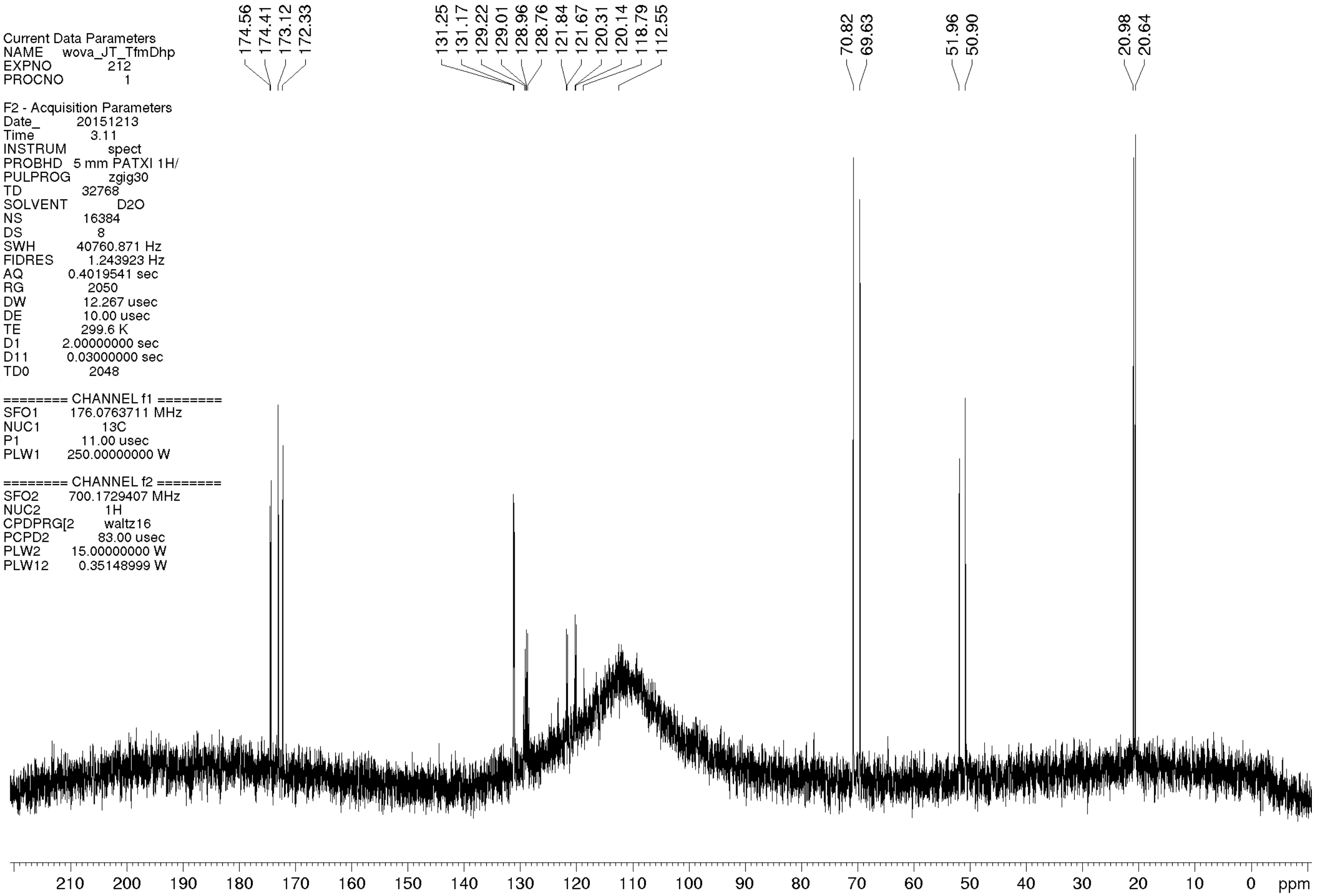

S20

^{19}F NMR spectrum of Ac-TfmDhp-O$^-$ in D_2O (buffer, pH 7)

Current Data Parameters
NAME wova_JT_TfmDhp
EXPNO 190
PROCNO 1

F2 - Acquisition Parameters
Date_ 20151212
Time 20.48
INSTRUM spect
PROBHD 5 mm PATXI 1H/
PULPROG zg
TD 65536
SOLVENT H2O+D2O
NS 1
DS 0
SWH 32894.738 Hz
FIDRES 0.501934 Hz
AQ 0.9961472 sec
RG 1290
DW 15.200 usec
DE 50.00 usec
TE 299.6 K
D1 1.00000000 sec
TD0 1

======== CHANNEL f1 ========
SFO1 658.7752138 MHz
NUC1 19F
P1 10.75 usec
PLW1 15.00000000 W

F2 - Processing parameters
SI 65536
SF 658.8180370 MHz
WDW EM
SSB 0
LB 1.00 Hz
GB 0
PC 10.00

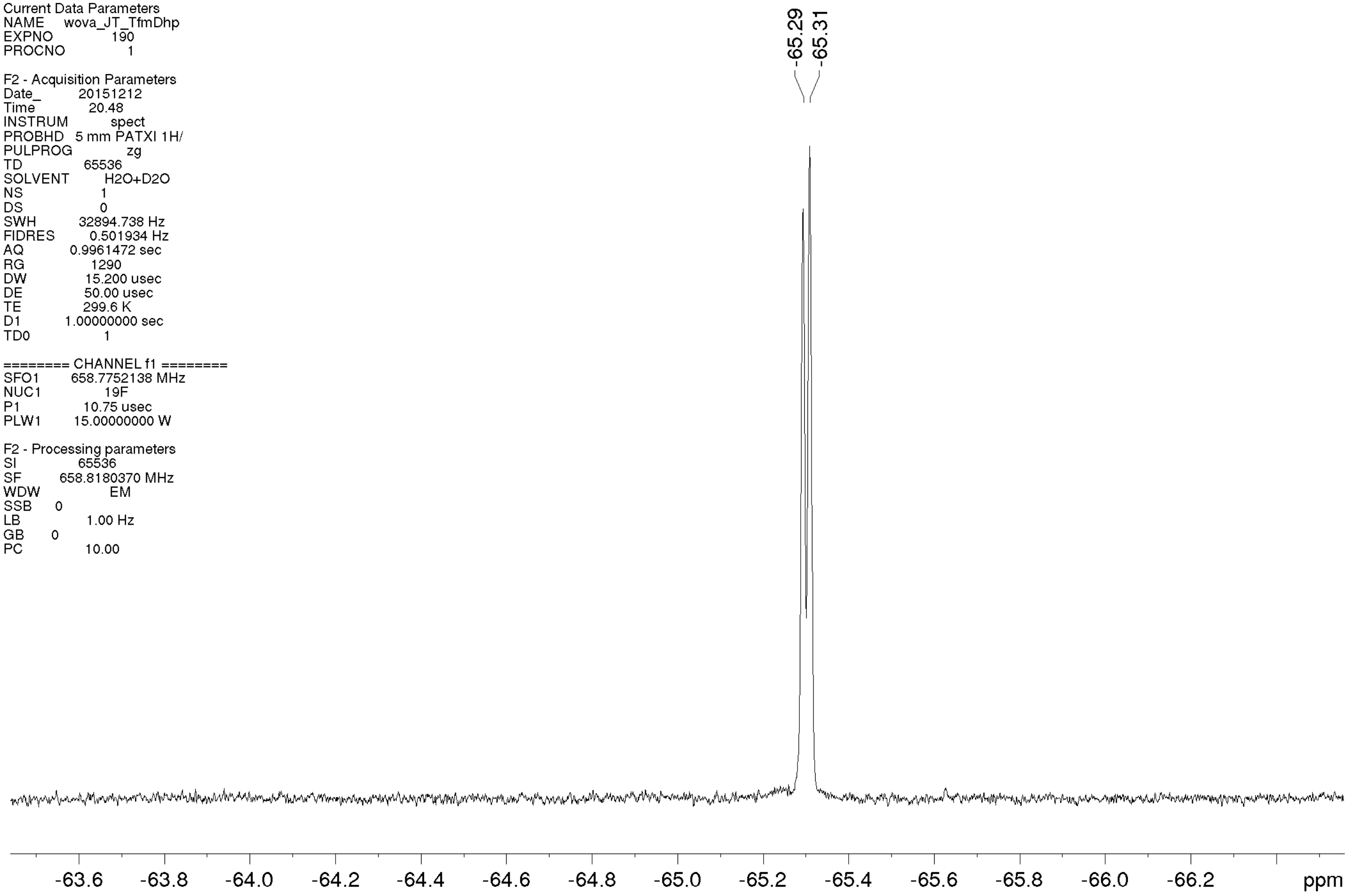

S21

^{1}H NMR spectrum of compound **6** (Ac-Dhp-OMe) in D_2O

Current Data Parameters
NAME wova_Ac-Dhp-OMe
EXPNO 70
PROCNO 1

F2 - Acquisition Parameters
Date_ 20151117
Time 10.46
INSTRUM spect
PROBHD 5 mm PATXI 1H/
PULPROG zg
TD 65536
SOLVENT D2O
NS 1
DS 0
SWH 8333.333 Hz
FIDRES 0.127157 Hz
AQ 3.9321599 sec
RG 74.88
DW 60.000 usec
DE 10.00 usec
TE 298.0 K
D1 1.00000000 sec
TD0 1

======== CHANNEL f1 ========
SFO1 700.1732908 MHz
NUC1 1H
P1 10.00 usec
PLW1 15.00000000 W

F2 - Processing parameters
SI 65536
SF 700.1700000 MHz
WDW EM
SSB 0
LB 0.20 Hz
GB 0
PC 10.00

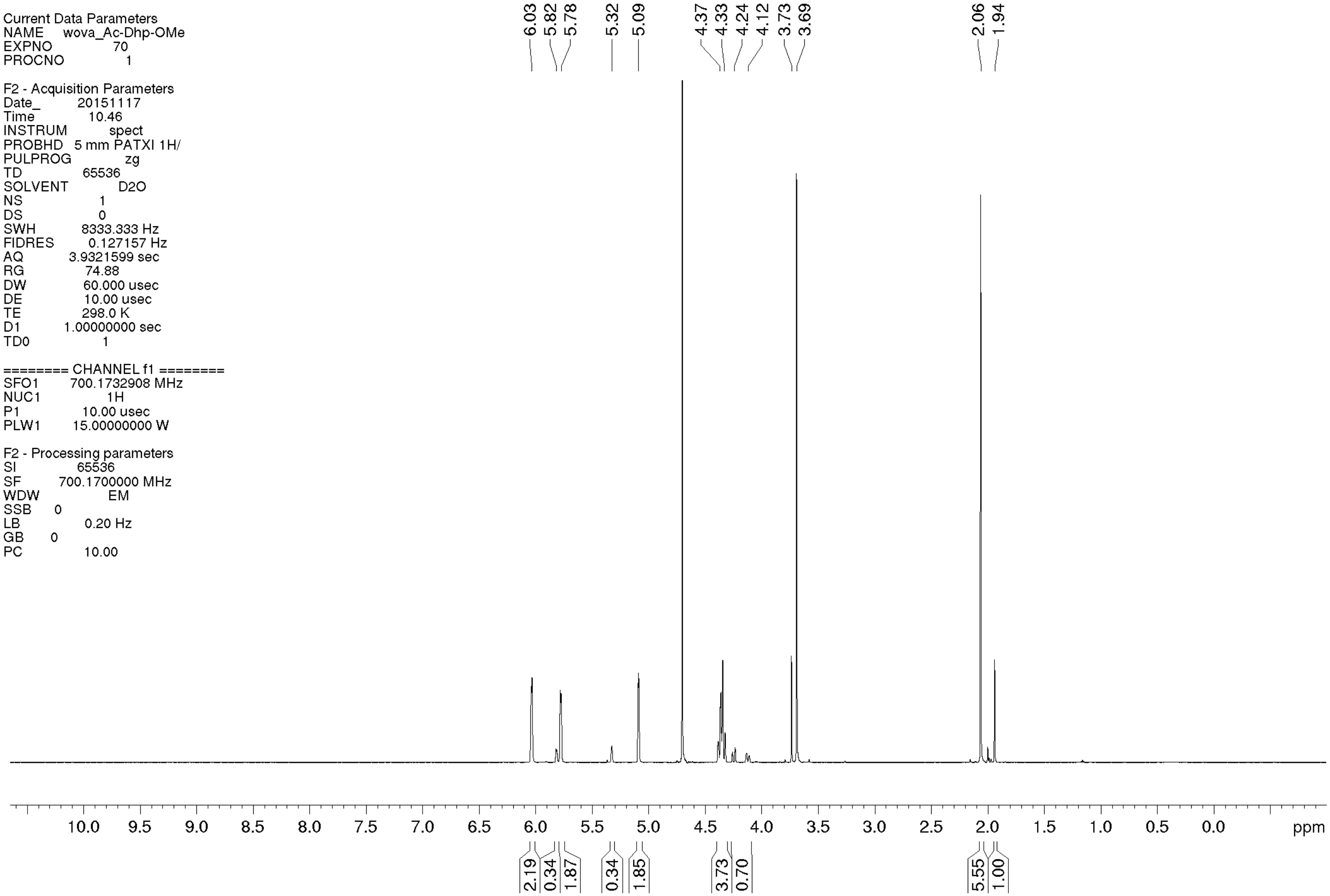

$^{13}C\{^1H\}$ NMR spectrum of compound **6** (Ac-Dhp-OMe) in D_2O

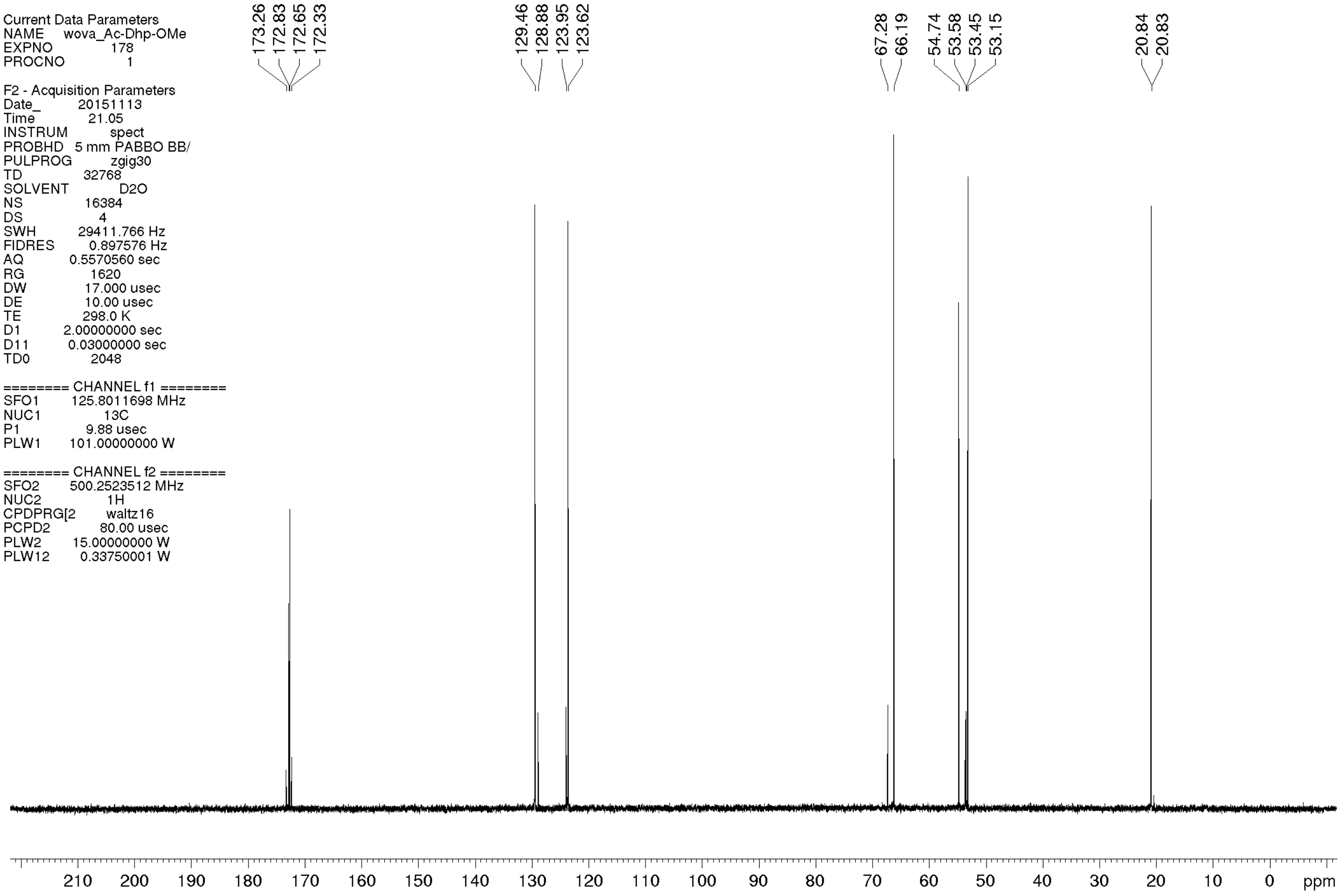

^{1}H NMR spectrum of Ac-Dhp-O^{-} in D_2O (buffer, pH 7)

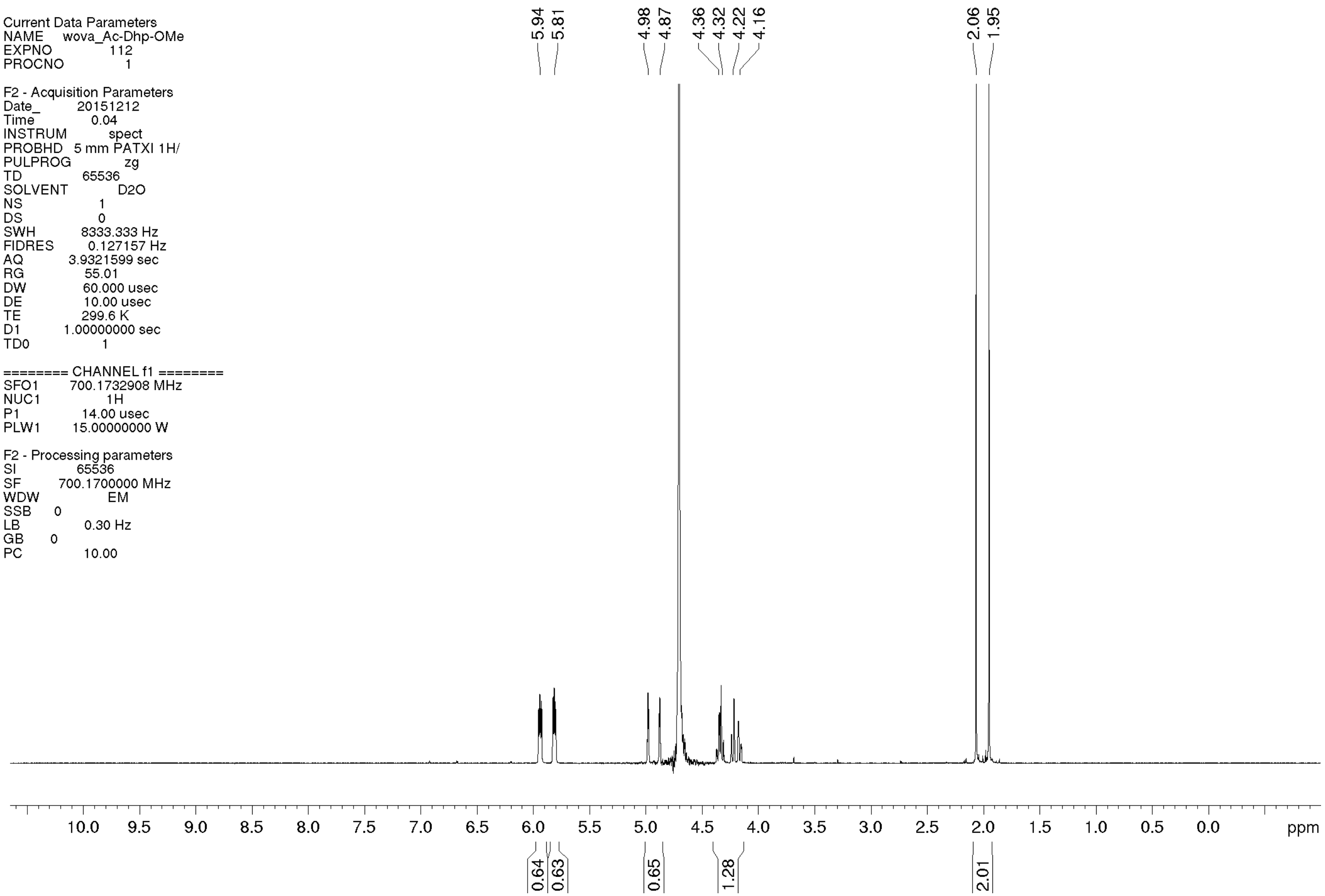

$^{13}C\{^1H\}$ NMR spectrum of Ac-Dhp-O$^-$ in D_2O (buffer, pH 7)

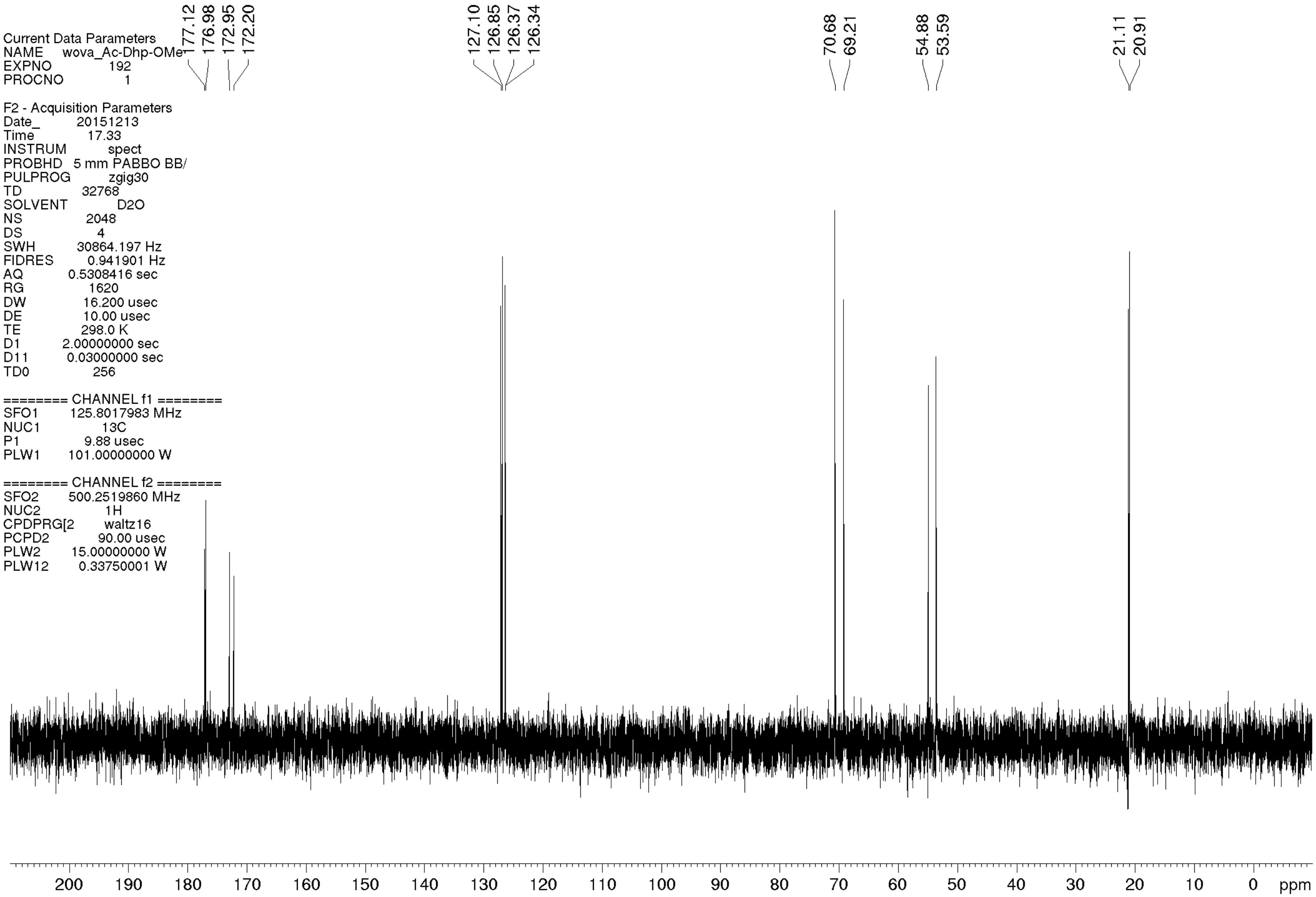

^{1}H NMR spectrum of HCl*TfmDhp-OMe in D_2O

Current Data Parameters
NAME wova_JT_TfmDhp
EXPNO 140
PROCNO 1

F2 - Acquisition Parameters
Date_ 20151210
Time 11.05
INSTRUM spect
PROBHD 5 mm PATXI 1H/
PULPROG zg
TD 65536
SOLVENT D2O
NS 1
DS 0
SWH 8333.333 Hz
FIDRES 0.127157 Hz
AQ 3.9321599 sec
RG 194.19
DW 60.000 usec
DE 10.00 usec
TE 299.6 K
D1 2.00000000 sec
TD0 1

======== CHANNEL f1 ========
SFO1 700.1732898 MHz
NUC1 1H
P1 11.00 usec
PLW1 15.00000000 W

F2 - Processing parameters
SI 32768
SF 700.1700000 MHz
WDW EM
SSB 0
LB 0.30 Hz
GB 0
PC 10.00

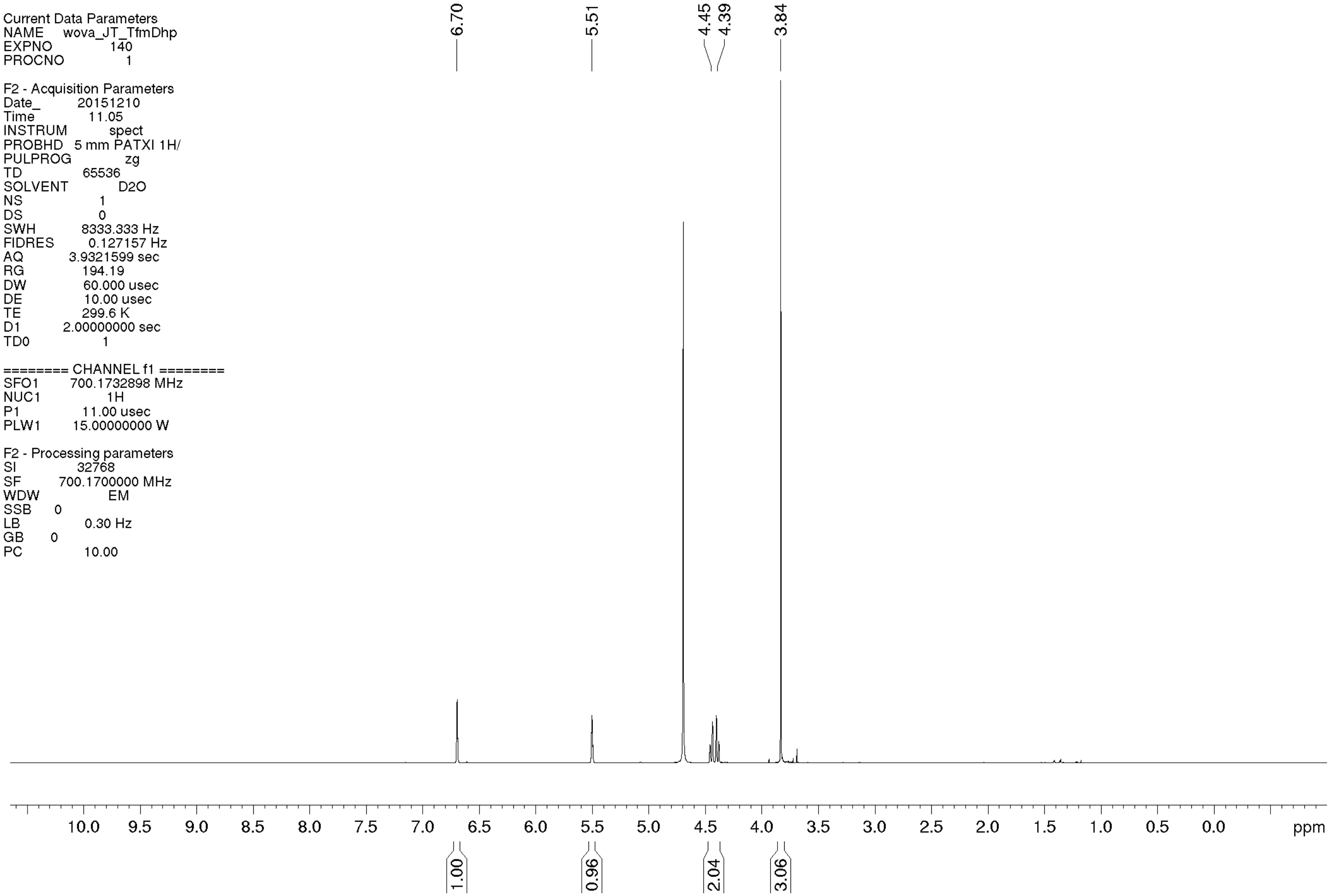

^{19}F NMR spectrum of HCl*TfmDhp-OMe in D_2O

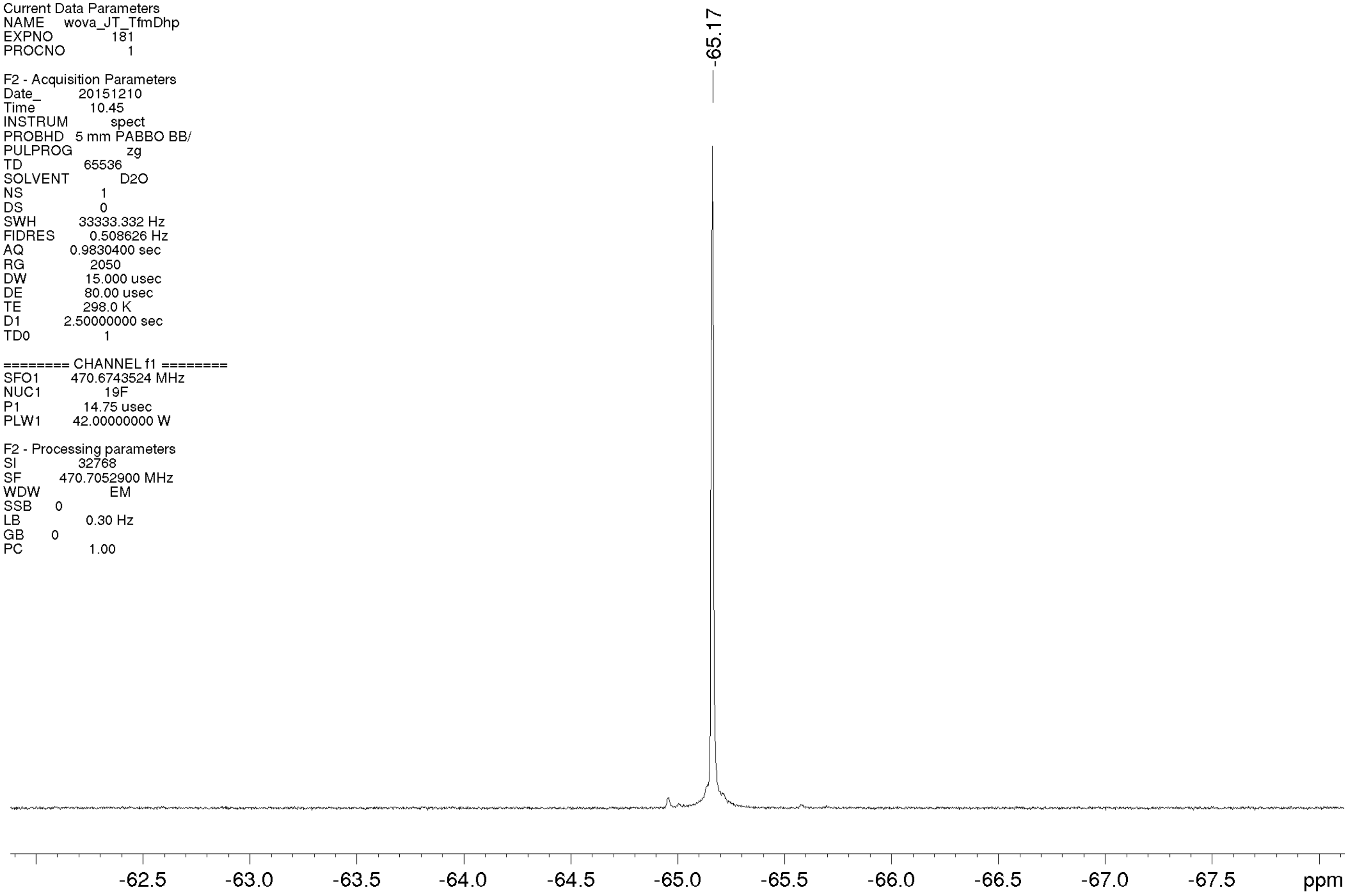

^{1}H NMR spectrum of compound **8** (Ac-TfmDhp-OMe) in D_2O

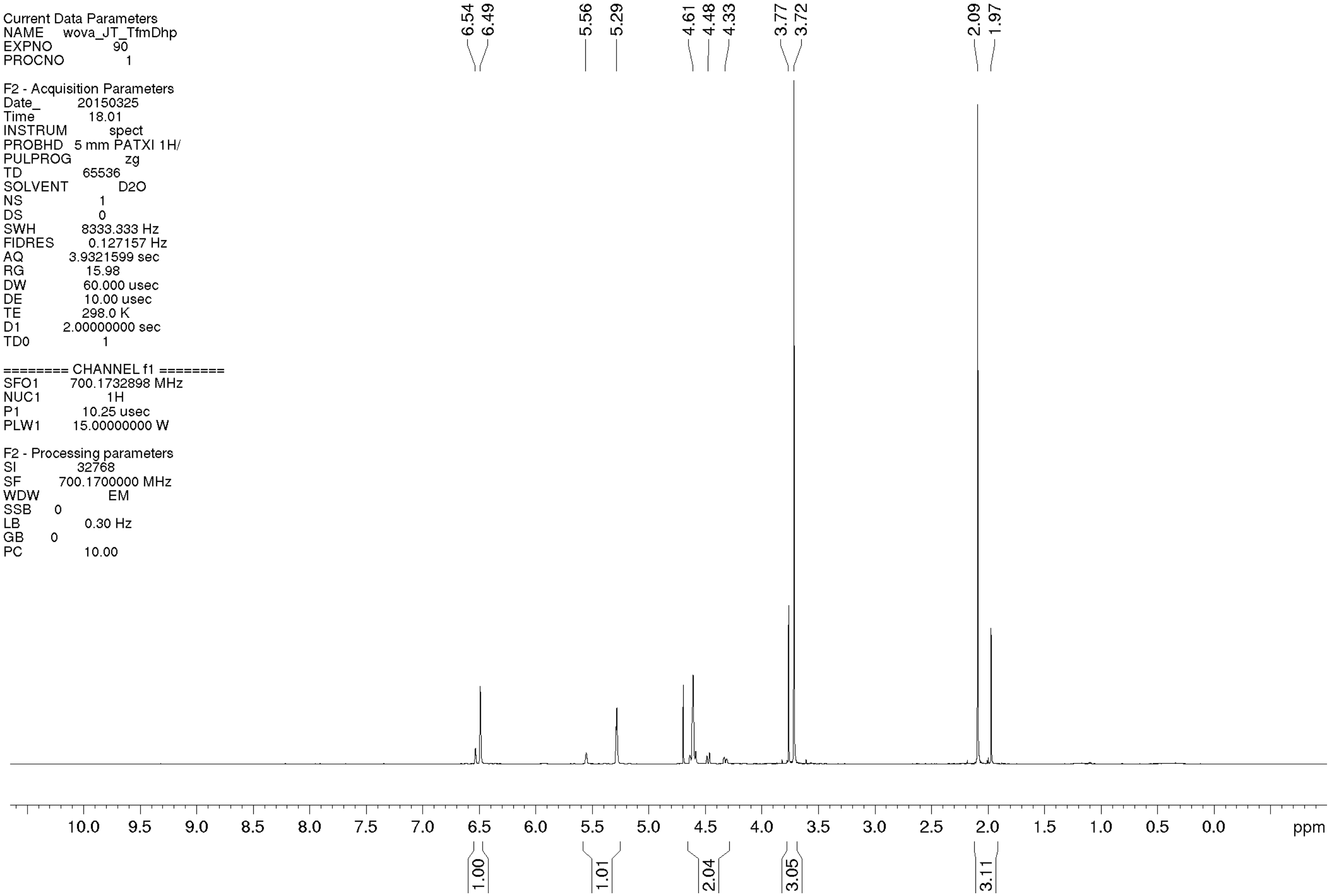

$^{13}C\{^1H\}$ NMR spectrum of compound **8** (Ac-TfmDhp-OMe) in D_2O

Current Data Parameters
NAME wova_JT_TfmDhp
EXPNO 95
PROCNO 1

F2 - Acquisition Parameters
Date_ 20150325
Time 18.50
INSTRUM spect
PROBHD 5 mm PATXI 1H/
PULPROG zgig30
TD 32768
SOLVENT D2O
NS 1024
DS 8
SWH 40760.871 Hz
FIDRES 1.243923 Hz
AQ 0.4019541 sec
RG 2050
DW 12.267 usec
DE 10.00 usec
TE 298.0 K
D1 2.00000000 sec
D11 0.03000000 sec
TD0 128

======== CHANNEL f1 ========
SFO1 176.0763711 MHz
NUC1 13C
P1 11.00 usec
PLW1 250.00000000 W

======== CHANNEL f2 ========
SFO2 700.1728007 MHz
NUC2 1H
CPDPRG[2 waltz16
PCPD2 65.00 usec
PLW2 15.00000000 W
PLW12 0.35148999 W

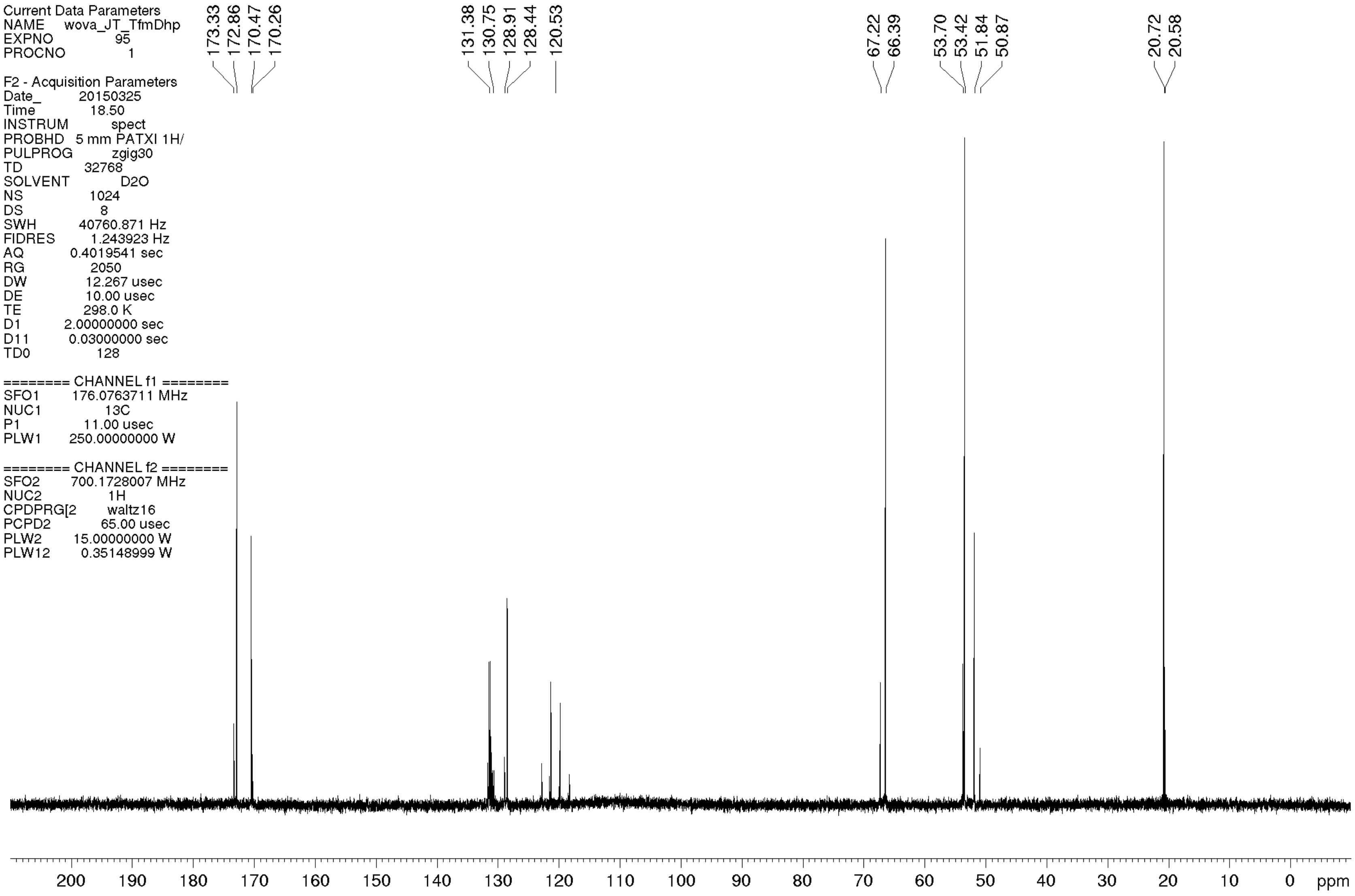

^{19}F NMR spectrum of compound **8** (Ac-TfmDhp-OMe) in D_2O

Current Data Parameters
NAME wova_JT_TfmDhp
EXPNO 81
PROCNO 1

F2 - Acquisition Parameters
Date_ 20150324
Time 14.25
INSTRUM spect
PROBHD 5 mm PATXI 1H/
PULPROG zg30
TD 65536
SOLVENT D2O
NS 1
DS 0
SWH 32894.738 Hz
FIDRES 0.501934 Hz
AQ 0.9961472 sec
RG 1290
DW 15.200 usec
DE 50.00 usec
TE 298.0 K
D1 1.00000000 sec
TD0 1

======== CHANNEL f1 ========
SFO1 658.7719197 MHz
NUC1 19F
P1 10.75 usec
PLW1 15.00000000 W

F2 - Processing parameters
SI 65536
SF 658.8180370 MHz
WDW EM
SSB 0
LB 1.00 Hz
GB 0
PC 10.00

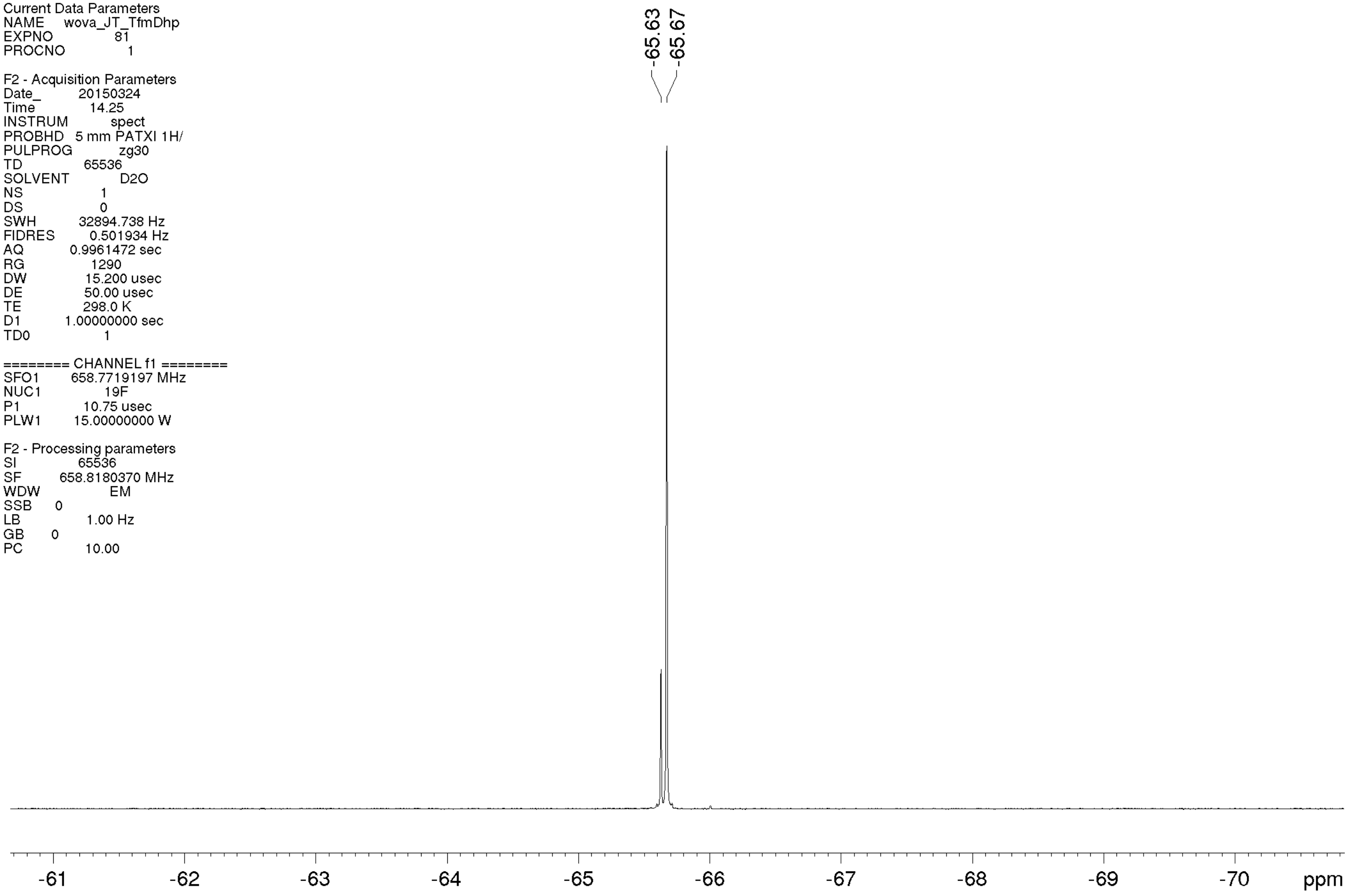

X-ray crystal structure of compound **5**, ellipsoid plot

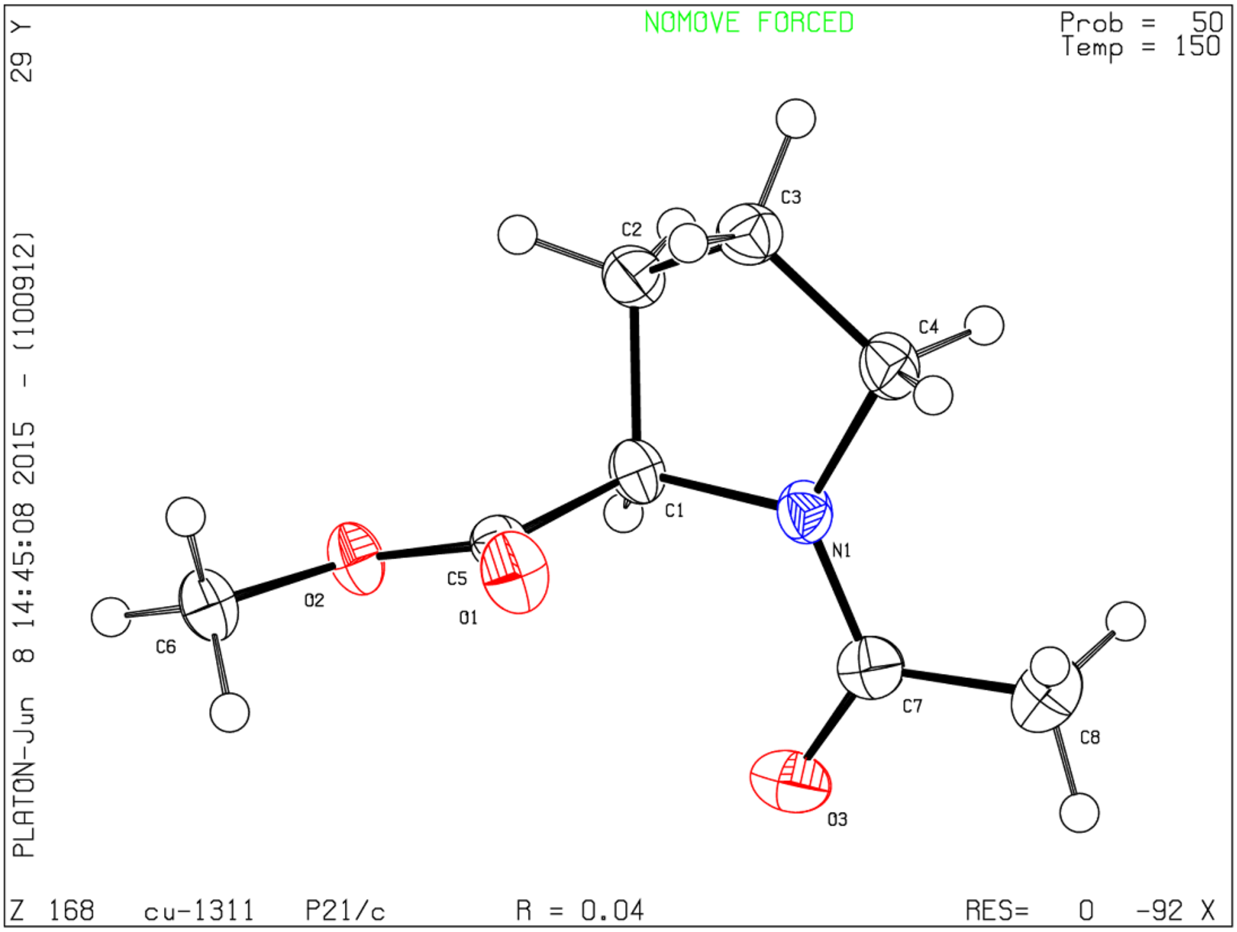

X-ray crystal structure of compound **6**, ellipsoid plot

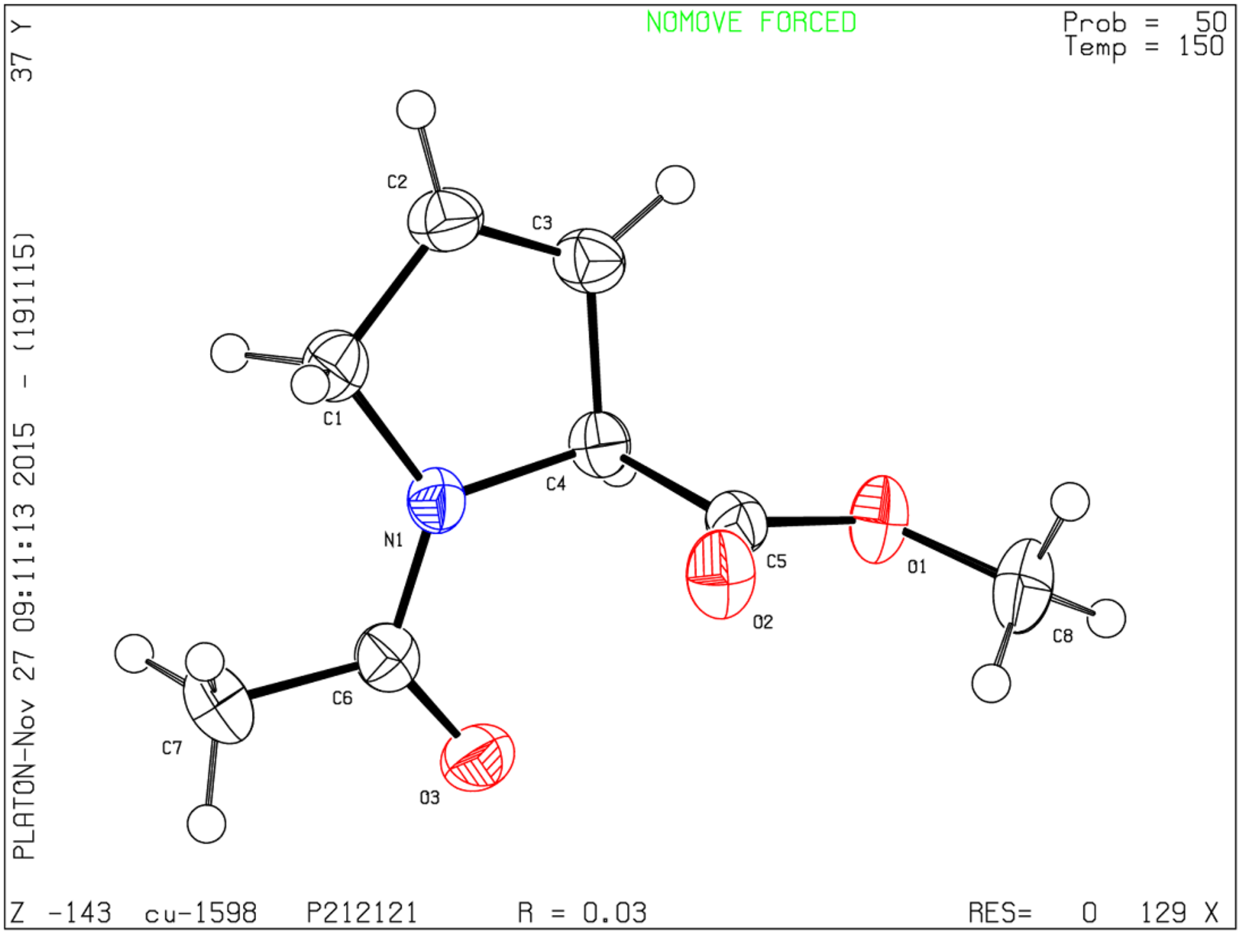

X-ray crystal structure of compound **8**, ellipsoid plot

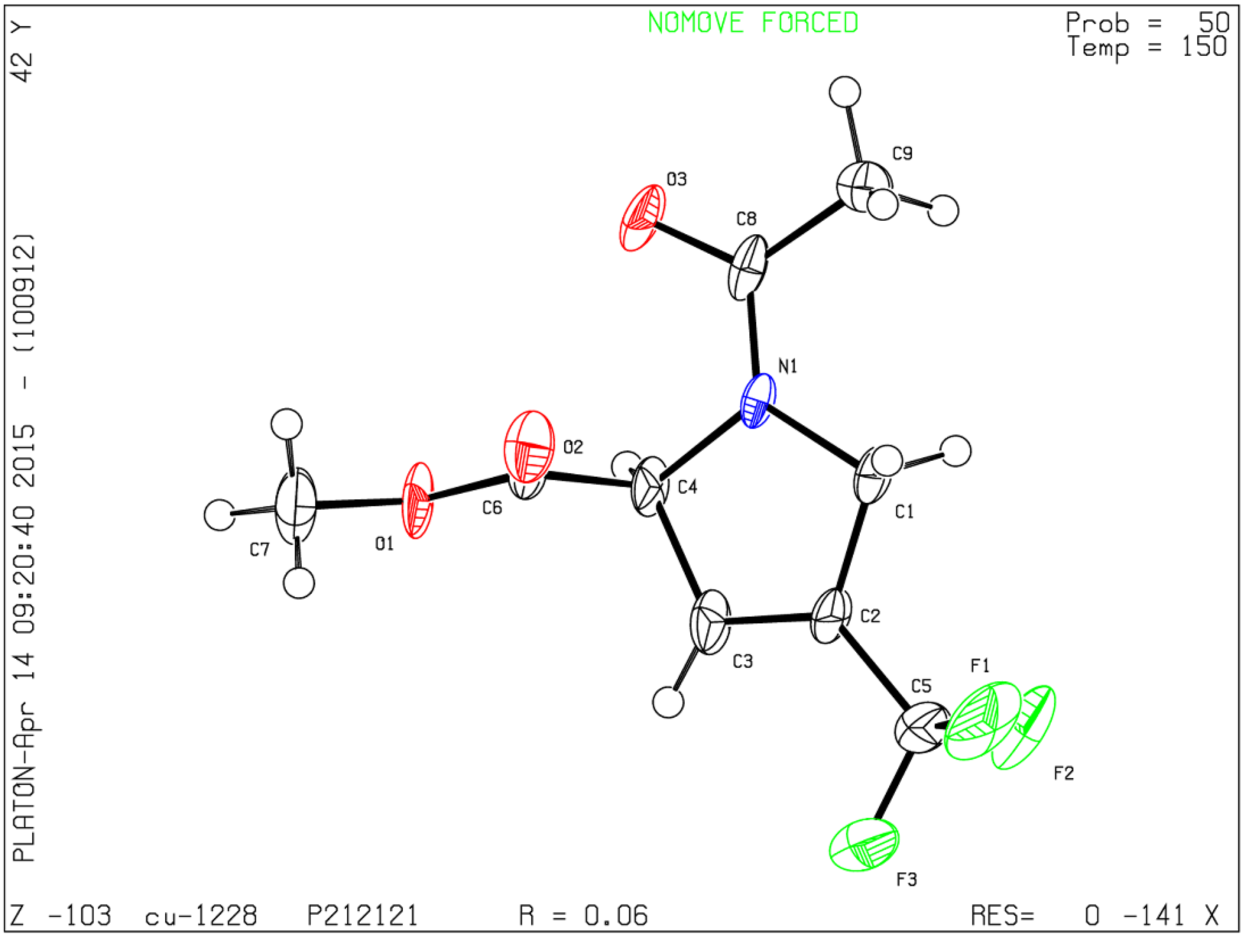

X-ray crystal structure of compound **9**, ellipsoid plot

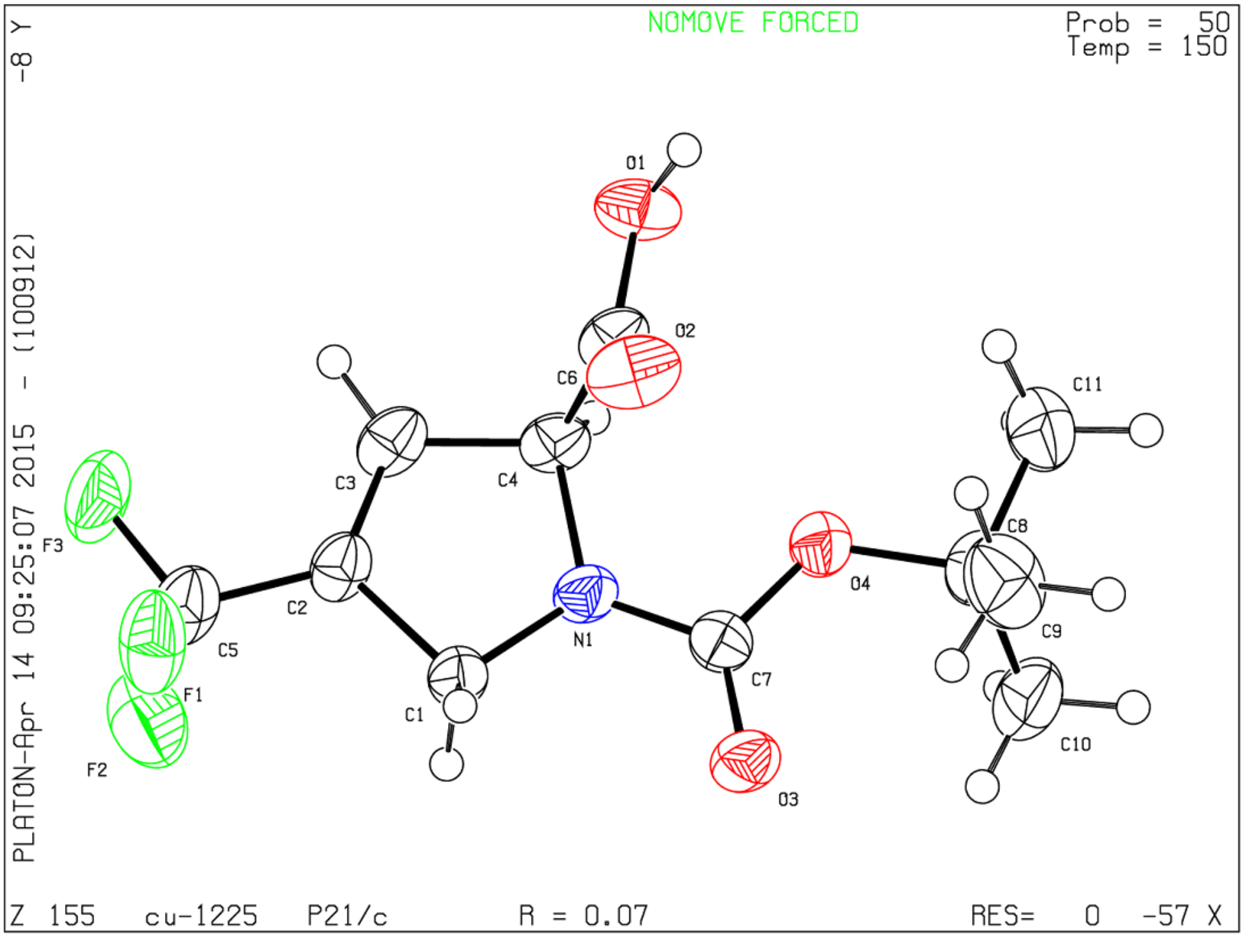

Supplement: File 1 — Experimental procedures, values for the amide rotational barriers in different solvents, copies of the NMR spectra and ellipsoid diagrams of the X-ray crystal structures. [file Beilstein_J_Org_Chem-12-589-s001.pdf]
